# Extracellular vesicles from UTX-knockout endothelial cells boost neural stem cell differentiation in spinal cord injury

**DOI:** 10.1186/s12964-023-01434-4

**Published:** 2024-02-29

**Authors:** Yudong Liu, Zixiang Luo, Yong Xie, Yi Sun, Feifei Yuan, Liyuan Jiang, Hongbin Lu, Jianzhong Hu

**Affiliations:** 1 Department of Spine Surgery and Orthopaedics, Xiangya Hospital, Central South University, Changsha, China; 2 Department of Sports Medicine, Xiangya Hospital, Central South University, Changsha, China; 3 Key Laboratory of Organ Injury, Aging and Regenerative Medicine of Hunan Province, Changsha, China; 4 Hunan Engineering Research Center of Sports and Health, Changsha, China; 5 National Clinical Research Center for Geriatric Disorders, Xiangya Hospital, Central South University, Changsha, China

**Keywords:** Epigenetics, Extracellular vesicles, Neural Differentiation, Spinal cord injury, UTX

## Abstract

**Background:**

Vascular endothelial cells are pivotal in the pathophysiological progression following spinal cord injury (SCI). The UTX (Ubiquitously Transcribed Tetratripeptide Repeat on Chromosome X) serves as a significant regulator of endothelial cell phenotype. The manipulation of endogenous neural stem cells (NSCs) offers a compelling strategy for the amelioration of SCI.

**Methods:**

Two mouse models were used to investigate SCI: NSCs lineage-traced mice and mice with conditional UTX knockout (UTX KO) in endothelial cells. To study the effects of UTX KO on neural differentiation, we harvested extracellular vesicles (EVs) from both UTX KO spinal cord microvascular endothelial cells (SCMECs) and negative control SCMECs. These EVs were then employed to modulate the differentiation trajectory of endogenous NSCs in the SCI model.

**Results:**

In our NSCs lineage-traced mice model of SCI, a marked decrease in neurogenesis was observed post-injury. Notably, NSCs in UTX KO SCMECs mice showed enhanced neuronal differentiation compared to controls. RNA sequencing and western blot analyses revealed an upregulation of L1 cell adhesion molecule (L1CAM), a gene associated with neurogenesis, in UTX KO SCMECs and their secreted EVs. This aligns with the observed promotion of neurogenesis in UTX KO conditions. In vivo administration of L1CAM-rich EVs from UTX KO SCMECs (KO EVs) to the mice significantly enhanced neural differentiation. Similarly, in vitro exposure of NSCs to KO EVs resulted in increased activation of the Akt signaling pathway, further promoting neural differentiation. Conversely, inhibiting Akt phosphorylation or knocking down L1CAM negated the beneficial effects of KO EVs on NSC neuronal differentiation.

**Conclusions:**

In conclusion, our findings substantiate that EVs derived from UTX KO SCMECs can act as facilitators of neural differentiation following SCI. This study not only elucidates a novel mechanism but also opens new horizons for therapeutic interventions in the treatment of SCI.

Video Abstract

**Graphical Abstract:**

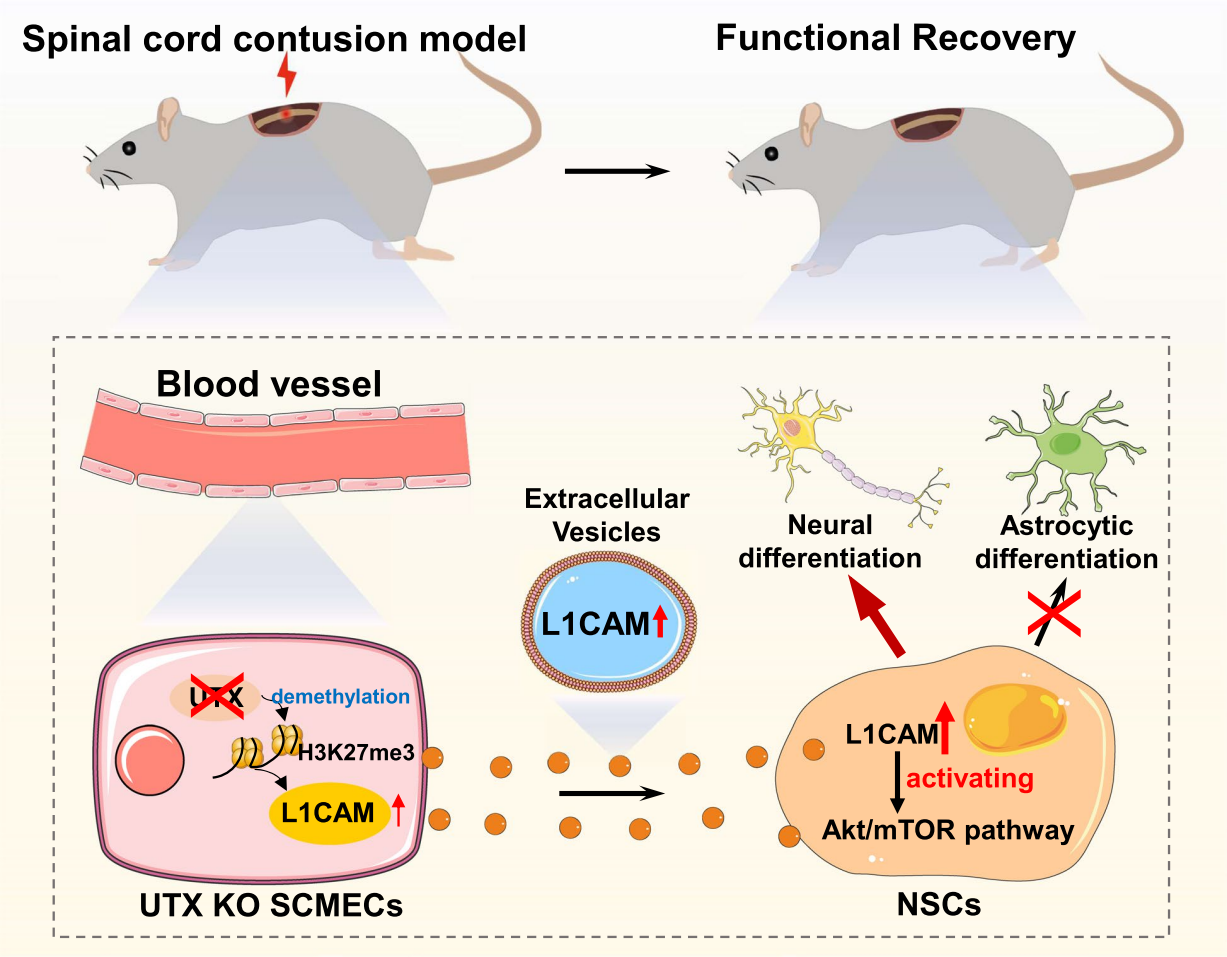

**Supplementary Information:**

The online version contains supplementary material available at 10.1186/s12964-023-01434-4.

## Background

Spinal cord injury (SCI) leads to major motor and sensory dysfunction and mainly affects young to middle-aged individuals, imposing significant economic burdens. Yet, effective treatments are still limited [1].

Secondary SCI exacerbates neural dysfunction, typically exceeding primary injury damage. It induces an inhibitory microenvironment, intensified by inflammatory cells like leukocytes and macrophages, which amplify inflammation [2, 3]. The pathophysiology of involves cell death, axonal degeneration, demyelination, glial scar formation, and inflammation, among other anomalies. The imbalance of these factors promoting and inhibiting recovery, influenced by these pathological events, hinders neural plasticity and functional recovery.

Historically thought to occur only in the brain, the discovery of neural stem cells (NSCs) in the adult spinal cord offers new prospects for non-invasive SCI treatments. However, the potential of these multipotent stem cells is seemingly restricted to the spinal cord’s ependymal cell group [4]. After SCI, NSCs undergo expansion, migration, and differentiation [5]. Notably, ependymal cells differentiate, primarily into glial cells and less frequently into neurons, despite abundant NSCs in the affected area [6]. Factors like myelin-associated glycoprotein (MAG) and chondroitin sulfate proteoglycans (CSPGs) promote glial over neural differentiation of NSCs [7]. Limited neural differentiation of NSCs restricts CNS regeneration post-injury. Thus, promoting NSCs’ neuronal differentiation is key for SCI repair.

NSCs reside in ‘stem cell niches,’ which regulate their renewal and differentiation, with blood vessels playing a key role in facilitating interactions with vascular endothelial cells [8]. Vascular endothelial cells in stem cell niches significantly affect neurogenesis, either by secreting factors or forming vessels that transport these factors into the CNS [9]. Studies of the brain’s subventricular zone (SVZ) underscore the importance of vascular signals, noting the close proximity of NSCs’ progeny to blood vessels [10]. Co-culturing endothelial cells with NSCs in vitro fosters neural differentiation and limits astrocytic differentiation [11, 12]. Similarly, co-transplanting these cells in vivo enhances NSCs proliferation and accelerates their neural differentiation [13]. Vascular endothelial cells may influence NSCs differentiation via Hairy/Enhancer of Split 6 (Hes6), encouraging neural fate over astrocytic differentiation [14].

Epigenetic research has pinpointed UTX (Ubiquitously Transcribed Tetratripeptide Repeat on Chromosome X) as a key histone demethylase in gene expression regulation. Studies reveal that UTX gene knockout (UTX KO) in spinal cord endothelial cells boosts vascular regeneration and recovery post-SCI [15], and directs macrophage polarization to the anti-inflammatory M2 subtype, aiding neurological recovery [16].

The L1 cell adhesion molecule (L1CAM), crucial in neural development, is linked to various neurological dysfunctions when aberrantly expressed [17–19]. It’s shown to favor neural over glial differentiation in neural precursors [20]. L1CAM has also been shown to encourage neural differentiation and inhibit glial differentiation of neural precursor cells in vitro [21], with its potential being explored in both normal and diseased neural contexts [22–25], and though minimal in healthy vascular endothelial cells, it escalates during tumors, injuries, and inflammation [26].

Extracellular vesicles (EVs) are key in intercellular communication and emerging as therapeutic agents. The disruption of blood vessels post-SCI underscores the therapeutic potential of EVs derived from damaged endothelial cells [27]. UTX KO in spinal cord microvascular endothelial cells (SCMECs) post-SCI facilitates macrophage polarization to the M2 subtype through EVs [16]. These EVs from endothelial cells can increase NSCs proliferation and reduce their astrocytic differentiation in vitro [28]. EVs from endothelial cells also promote NSCs proliferation and migration in acute ischemic brain injury, improving outcomes [29]. Proteomic analyses have identified L1CAM in EVs across various cell types, including those from spinal cord tissue [30–34].

In this study, we highlight the marked upregulation of L1CAM in UTX KO SCMECs, as revealed by RNA sequencing. Our findings indicate that UTX KO epigenetically enhances L1CAM expression in SCMECs post-SCI, influencing NSCs differentiation towards neuronal pathways.

## Methods

### Mice

All animal experimental protocols were approved by the Ethics Committee of Central South University (CSU) for scientific research. The conditional SCMECs UTX KO mice were generated by Tek-Cre mice (Shanghai Model Organisms, SJ-008863) mating with UTX^flox/flox^ mice(Jackson Laboratory, stock no. 021926). Their offsprings were intercrossed to generate Tek-Cre (mice expressing Cre recombinase driven by Tek promoter)-UTX^flox/flox^ mice (SCMECs UTX KO mice). The littermates of UTX^flox/flox^ mice acted as a negative control (NC mice).

The NSCs lineage-traced mice were generated by Nestin-CreER^T2^ mice (Jackson Laboratories, stock no. 016261) mating with Rosa26-STOP-tdTomato mice (Jackson Laboratories, stock no. 007909). Their offsprings were intercrossed to generate Nestin-CreER^T2^-Rosa26-STOP-tdTomato mice (female mice expressing Cre recombinase with tamoxifen by the Nestin promoter; Rosa26-STOP-tdTomato). 6-week-old NSCs lineage-traced mice were intraperitoneally injected with tamoxifen in corn oil at a dose of 10 μl 10 μg/μl qd for 5 consecutive days. After tamoxifen intervention, NSCs will spontaneously emit red fluorescence. Spinal cord injury was modeled when the mice were 8 weeks old.

### Establishment of the contusion SCI model

8-week-old mice were anesthetized. After laminectomy at T10, moderate contusion injury of the spinal cord was induced by a modified Allen’s weight drop apparatus (10 g weight at a vertical height of 20 mm). Mice in the sham group were subjected to laminectomy without contusion. Bladders were manually massaged twice daily until full voluntary or autonomic voiding was obtained, and antibiotic (penicillin sodium, Solarbio, P8420) was administered once daily for 3 days post-surgery.

### Spinal cord microvascular endothelial cells isolation

SCMECs were isolated from the spinal cord of 8-week-old mice. After euthanizing the mice, the entire spine was take out sterility. Inject cold PBS (Solarbio, P1020) through the sacral opening of the spine to expel the entire spinal cord from the spine. After removing the dura mater and cutting the spinal cord tissue, the tissue was digested using 0.1% collagenase II (Gibco, 17101015). Centrifuge at 4000 rpm in 20% BSA (Meilunbio, MB4219) for myelin removal. Next, digest the tissue with 0.1% collagenase/dispersase (Roche, 10269638001). After washing with complete culture medium (EGM-2, Lonza, CC-4176), pellets were re-suspended in complete culture medium with 5% FBS (Opcel, BS-1105) and plated onto T25 flask coated with collagen I rat tail (Gibco,10483-01) for further experiments.

### Culture of NSCs and L1CAM knockdown

NSCs were isolated from the wild-type (Purchased from Charles River) neonatal mice. Brains were detached. Remove meninges and large vessels under microscope. Dissected tissues were digested by 0.05% Trypsin–EDTA (Gibco, 25300054) for 15 min and centrifuged. The pellets were filtrated with 75 μm sieve and resuspended in mouse NSC culture medium (Cyagen, MUXNF-90011), seeded in T-75 culture flasks, and incubated at 37 °C and 5% CO_2_ for 5 days until neurospheres appeared.

For knockdown of L1CAM, neurospheres were dissociated into single cells and infected with L1CAM shRNA recombinant adenovirus (Genechem, China). Scrambled shRNA adenovirus was used as control.

### Evaluation of the integrity of the plasma membrane of SCMECs

The integrity of the plasma membrane (PM) of SCMECs was determined using the Evans Blue dye (EB, Sigma, E2129) permeability method [35]. Damage to the PM of SCMECs can lead to the degradation of the tight junction protein on the PM, which in turn leads to the destruction of the blood-spinal cord barrier [36]. EB will leak from the blood vessels into the extracellular matrix. EB dye presents red fluorescence under a fluorescence microscope. Sham group mice and mice 3 days post-SCI were injected with 0.2 ml 2% EB through the tail vein. 1 h later, the spinal cord tissues were removed after perfusion. Frozen slicing the spinal cord tissues, observe them under a fluorescence microscope.

### In vitro differentiation of NSCs

For NSCs differentiation, NSCs digested into single cells were seeded on poly-D-lysine-coated (Sigma, P4832) slides in cell culture plates and grown with different differentiation media for 5 days. For neuronal differentiation, NSCs were cultured in neuronal differentiation medium composed of neurobasal medium (Gibco, 21103049), 2% B-27 (Gibco, A1486701), 2 mM L-glutamine (Gibco, A2916801), 2 μM all-trans retinoic acid (Sigma, R2625), 5 μM forskolin (MCE, HY-15371) and 0.05 g/l penicillin/streptomycin (Gibco, 15140148). For astrocytic differentiation, NSCs were cultured in NSC culture medium with 5% FBS. For oligodendrocytic differentiation, NSCs were cultured in oligodendrocyte differentiation medium containing DMEM-F12 (Gibco, 10565018), 2% B-27 and 200 ng/ml insulin-like growth factor 1 (Cyagen, REGFP-09011).

### Intervention of SCMECs culture supernatant on differentiation of NSCs

When UTX KO SCMECs and NC SCMECs grow to the point where they are about to converge, replace the culture medium with serum-free medium (EGM-2 adding cytokines in addition to FBS) and continue to culture. 1 day later, collect the supernatant separately and add essential factors 2% B-27, 2 mM L-glutamine, and 2 μM all-trans retinoic acid and 5 μM forskolin as the supernatant for inducing neuronal differentiation (neuron-differentiation supernatant). Add 5% FBS to the neuron-differentiation supernatant as the inducing astrocytic differentiation supernatant (astrocyte-differentiation supernatant).

### Co-culture of SCMECs and NSCs

To investigate the effect of inhibiting the secretion of UTX KO SCMECs-derived EVs (KO EVs) or NC SCMECs-derived EVs (NC EVs) on NSCs differentiation, we need to co-culture SCMECs and NSCs. This study used a 24-well plate with 0.4 μm transwell chamber (Corning, 3413) to establish a co-culture system for SCMECs and NSCs. The SCMECs were seeded in the upper chamber of the transwell. In order to inhibit the secretion of SCMECs EVs, the upper complete medium of the experimental group contained 0.01% GW4869 (Sigma, D1692, GW4869 group), while the control group did not contain GW4869. GW4869 is an inhibitor of sphingomyelinase which can inhibit the biogenesis and release of EVs [37]. NSCs were digested into single cells and seeded in the lower chamber to construct the SCMECs-NSCs co-culture system.

### Isolation and identification of the EVs derived from SCMECs

KO EVs and NC EVs were isolated by differential centrifugation, as reported in previous studies [38]. Transmission electron microscopy (TEM; Hitachi, JPN) was used to identify the morphology of EVs. Nanoparticle tracking analysis (NTA) was used to measure EVs diameter and particle number.

### Immunoelectron microscopy

20uL of the resuspended samples were added dropwise to 200-mesh grids and incubated at room temperature for 10 min, then the grids were negatively stained with 2% phosphotungstic acid for 3 min, and the remaining liquid was removed by filter paper. Then observed with a JEM1400 transmission electron microscope.

### PKH67-labeled SCMECs-derived EVs

SCMECs-derived EVs were labeled with a green fluorescent lipophilic dye PKH67 (Solarbio, D0031) to monitor the motion of the EVs. In brief, after EVs were incubated with 5 µM PKH67 dyeing working solution for 5 min. The labeled EVs were washed twice and resuspended in sterile PBS. Then, they could be used for subsequent in vivo and in vitro experiments.

### RNA sequencing analysis

For RNA-Sequencing analysis, the Aksomics Corporation constructed the library and performed the sequencing (Aksomics, China). Briefly, UTX KO SCMECs and NC SCMECs total RNA were extracted using Trizol reagent (Invitrogen, 15596026). Total RNA samples were enriched by oligo dT and then KAPA Stranded RNA-Seq Library Prep Kit (Illumina, USA) was used to construct the library, followed by sequencing using an Illumina NovaSeq 6000 sequencer (Illumina, USA). Each group contains three biological replicates.

### Immunofluorescence analysis

Cells seeded on slides or frozen sections of spinal cord were fixed with 4% paraformaldehyde, washed with PBS 3 times, and permeabilized with 0.1% Triton X-100 (BioFroxx, 143306) in PBS. Then, the samples were blocked with 4% BSA in PBST (Solarbio, P1031) and incubated with specific primary antibodies (The antibodies used are listed in Table 1) at 4 °C overnight. The sections were washed three times with PBS, incubated with the secondary antibody, and then stained with DAPI (Genetex, GTX30920).

**Table 1:** List of antibodies used in this study

Name	Company	Catalog Number	Comments
anti-Nestin	Wako	012–26843	1:200 dilution (IF)
anti-NeuN	Abcam,	ab279296	1:400 dilution (IF)
anti-NeuN	Sigma	ABN78A4	1:100 dilution (IF)
anti-GFAP	Abcam	ab53554	1:800 dilution (IF) 1:1000 dilution (WB)
anti-Tuj-1	Biolegend	801202	1:200 dilution (IF) 1:1000 dilution (WB)
anti-L1CAM	Proteintech	67115-1-Ig	1:200 dilution (IF) 1:2000 dilution (WB)
anti-CD31	R&D	FAB3628G	1:200 dilution (IF)
anti-CD31	Proteintech	65058-1-Ig	1:100 dilution (IF)
anti-TSG101	Proteintech	28283-1-AP	1:200 dilution (IF)
anti-SOX2	Abcam	ab171380	1:200 dilution (IF)
anti-O4	Sigma	MAB345M	10 μg/mL (IF)
anti-Akt	Wanlei	WL0003b	1:500 dilution (WB)
anti-p-Akt	Proteintech	66444-1-Ig	1:5000 dilution (WB)
anti-mTOR	Wanlei	WL02477	1:500 dilution (WB)
anti-p-mTOR	Proteintech	67778-1-Ig	1:2000 dilution (WB)
Alexa Fluor® 488 Donkey Anti-Mouse IgG	Abcam	ab150105	1:800 dilution (IF)
Alexa Fluor® 647 Donkey Anti-Mouse IgG	Abcam	ab150107	1:800 dilution (IF)
Alexa Fluor® 488 Donkey Anti-Rat IgG	Abcam	ab150153	1:800 dilution (IF)
Alexa Fluor® 594 Donkey Anti-Rat IgG	Abcam	ab150156	1:800 dilution (IF)
Alexa Fluor® 594 Donkey Anti-Rabbit IgG	Abcam	ab150076	1:800 dilution (IF)
Alexa Fluor® 594 Donkey Anti-Goat IgG	Abcam	ab150136	1:800 dilution (IF)
Alexa Fluor® 488 Donkey Anti-Rabbit IgG	Abcam	ab150073	1:800 dilution (IF)
Alexa Fluor® 647 Donkey Anti-Goat IgG,	Abcam	ab150135	1:800 dilution (IF)
Alexa Fluor® 488 Donkey Anti-Goat IgG	Abcam	ab150129	1:800 dilution (IF)
Goat anti-rabbit IgG	Proteintech	SA00001-2	1:5000 dilution (WB)
Goat anti-mouse IgG	Proteintech	SA00001-1	1:5000 dilution (WB)
Rabbit anti-goat IgG	Proteintech	SA00001-4	1:5000 dilution (WB)

### Western blot

Extracting cellular proteins using RIPA (Solarbio, R0010) lysis method. The protein concentration was measured with a BCA protein quantitation kit (Thermo Scientific, 23225). Proteins were separated by 10% SDS-PAGE (L1CAM, mTOR and p-mTOR use 6% SDS-PAGE to isolate proteins) and then transferred to nitrocellulose membranes. The membranes were blocked and incubated with the primary antibody (The antibodies used are listed in Table 1) before secondary antibody incubation. Pierce™ ECL Plus Western blotting substrate was used to detect the proteins. ImageJ was used for quantitative identification.

### Evaluation of the locomotive function

The BMS (Basso Mouse Scale) was utilized before surgery and 1 day, 1, 2, 3, 4, 5 and 6 weeks after SCI to evaluate the motor function [39]. Each mouse was observed for 5 min, and the average BMS and sub-scoring were recorded by two trained researchers and blinded to the experimental design.

### Statistics

The results were statistically analyzed with SPSS 22.0 (SPSS, Inc.). All data were presented as the means ± standard deviation (SD). Statistical analysis of multiple-group comparison was performed by one-way analysis of variance (ANOVA), followed by the Bonferroni post hoc test. Values of *p* less than 0.05 were considered statistically significant.

## Results

### NSCs were activated after SCI and migrated to the edge of the injured area, and most of the migrated NSCs differentiated into astrocytes

**Fig. 1:**
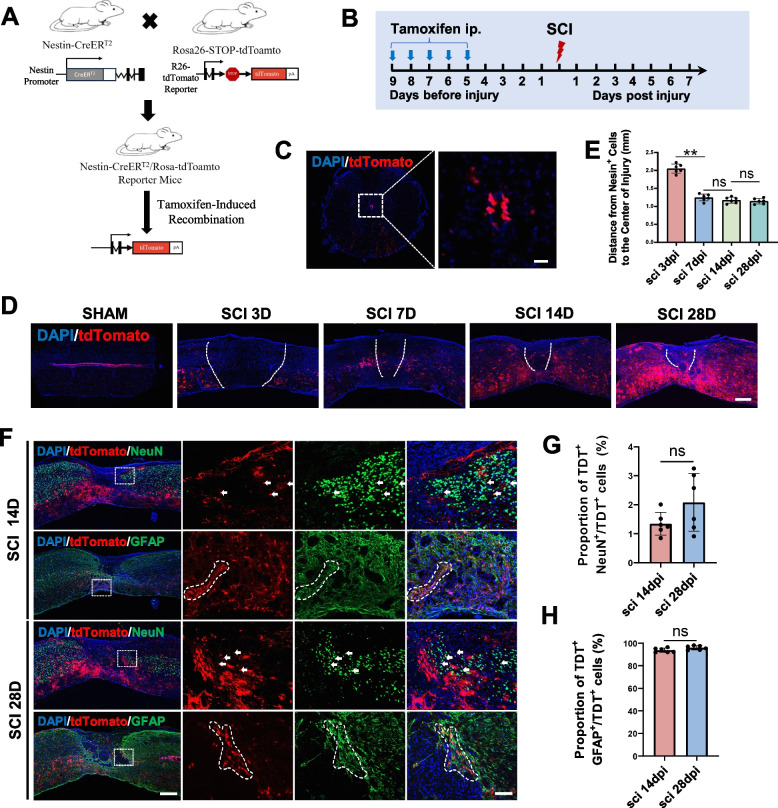
NSCs were activated and migrated to the injured area after SCI, and most of the migrated NSCs differentiated into astrocytes. **A** Construction of Nestin-CreER^T2^-Rosa26-STOP-tdTomato mice (NSCs lineage-traced mice). **B** The administration method of tamoxifen. **C** Immunofluorescence identification of NSCs (tdTomato, red) of spinal cord in NSCs lineage-traced mice. Scale bar, 20 μm. **D** Immunofluorescence analysis of the migration of NSCs (tdTomato, red) after SCI in NSCs lineage-traced mice, Scale bar, 100 μm. **E** Statistical analysis of the distance from tdTomato^+^ cells to the center of injury (mm) in figure D, *n* = 6 per group. **F** Immunofluorescence analysis of the differentiation of endogenous NSCs in NSCs lineage-traced mice at 14 and 28 days after SCI. NeuN is a neuronal marker and GFAP is a astrocytic marker. Scale bar, 100 μm and 40 μm. **G**, **H** Statistical analysis of the ratio of NeuN^+^tdTomato^+^ cells to tdTomato^+^ cells and the ratio of GFAP^+^tdTomato^+^ cells to tdTomato^+^ cells in figure F, *n* = 6 per group. ^ns^*P* > 0.05, *P < 0.05, **P < 0.01, compared with corresponding control group

Nestin, as an intermediate filament, has been used as a classical NSCs marker, refer to Shimada et.al [40]. However, NSCs will lose their Nestin phenotype after differentiation and even other cells will express Nestin after SCI [41]. So we chose Nestin-CreER^T2^-Rosa26-STOP-tdTomato mice to trace the fate of NSCs (Fig. 1A). This model allowed us to irreversibly label NSCs with red fluorescence upon tamoxifen injection, enabling the tracking of NSCs even after their differentiation into other cell types (Fig. 1B). Following SCI, endogenous NSCs were activated and migrated towards the periphery of the injured area. Utilizing Nestin-CreERT2-Rosa26-STOP-tdTomato mice as a lineage-traced model, we observed that the majority of these migrated NSCs differentiate into astrocytes.

To assess the temporal and spatial dynamics of NSCs, spinal cord tissues were collected from both the sham group and at various time points post-SCI: day 3, 7, 14, and 28. In sham group, NSCs were exclusively localized to the central canal of the spinal cord (Fig. 1C, Fig. S1A). Post-SCI, activated NSCs underwent morphological changes, transitioning from a rounded to a branched shape, and migrated outside the central canal (Fig. S1B). By the 3rd day post-injury, these cells were still en route to the damaged area. By the 7th day, most NSCs had reached the periphery of the injured site and remained there, with only a minority penetrating the core of the damaged area (Fig. 1D, E). We also observed a phenomenon where migrated NSCs always adhered to regenerated blood vessels on the 3rd day of SCI (Fig. S1C).

We further investigated the differentiation patterns of these activated NSCs. On the 3rd day post-SCI, neural differentiation was not yet observed, as indicated by the absence of tdTomato (TDT) signal expression for a neuronal marker. Approximately 40% of NSCs had differentiated into astrocytes at this stage. By the 7th day, over 90% of the migrated NSCs had differentiated into astrocytes, predominantly residing at the periphery of the injury site (Fig. S1D-F). From day 7, the differentiated astrocytes accounted for 30% of the total astrocytes and maintained this proportion (Fig. S1G). After 14 days, NSCs in the injured area had fully differentiated, almost exclusively into astrocytes, which contributed to the formation of a glial scar encircling the injury center (Fig. 1F-H).

### SCMECs UTX KO enhanced NSCs migration and neural differentiation post-SCI

#### Impact of SCMECs UTX KO on NSCs migration

**Fig. 2:**
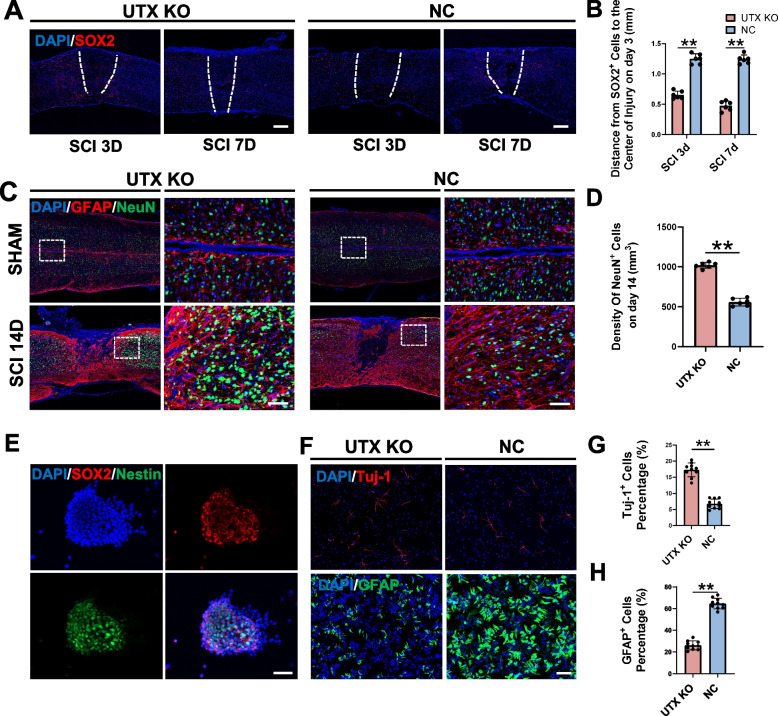
SCMECs UTX KO promoted the migration and neural differentiation of NSCs after SCI. **A** Immunofluorescence analysis of the migration of endogenous NSCs on the 3rd and 7th day after SCI in UTX KO and NC mice. Scale bar, 100 μm. **B** Statistical analysis of the distance from tdTomato^+^ cells to the center of injury (mm) in figure A, *n* = 6 per group. **C** Immunofluorescence analysis of spatial distribution of NeuN^+^ cells (neuron marker) and GFAP^+^ cells (astrocyte marker) in UTX KO and NC mice in the sham group and 14 days after SCI. Scale bar, 40 μm. **D** Statistical analysis of the density of NeuN^+^ cells in the injured area after 14 days of SCI (mm^2^) in figure C, *n* = 6 per group. **E** Nestin (NSC marker) and SOX2 (NSC marker) immunofluorescent identification of primary lsolated neurosphere formed by NSCs aggregation. Scale bar, 20 μm. **F** Immunofluorescent analysis of the neural differentiation and astrocytic differentiation of NSCs in vitro intervened by UTX KO SCMECs supernatant (UTX KO group) and NC SCMECs supernatant (NC group). Scale bar, 40 μm. **G**, **H** Statistical analysis of Tuj-1^+^ cells and GFAP^+^ cells to all cells in each group in figure F, *n* = 10 per group. ^ns^*P* > 0.05, *P < 0.05, **P < 0.01, compared with corresponding control group

Emerging evidence suggests that vascular-related factors play a pivotal role in neuroblast migration [42]. To investigate the influence of SCMECs UTX KO on NSCs migration, we utilized SRY (sex determining region Y)-box 2 (SOX2) as a marker for NSCs, given that Tek-Cre mice with SCMECs UTX KO are incompatible with Nestin-Cre lineage tracing. We focused on the 3rd and 7th day post-SCI, critical time points for NSCs migration. Our findings revealed that SOX2^+^ cells in UTX KO mice were more proximal to the injury center on both the 3rd and 7th days post-SCI, suggesting that UTX KO in SCMECs facilitated NSCs migration (Fig. 2A, B).

#### Differentiation patterns in UTX KO and NC mice

We performed an indirect comparison of NSCs differentiation between UTX KO and NC mice by labeling neurons and astrocytes. There was no significant difference in the spatial distribution and density of neurons in the uninjured spinal cord between the two groups (Fig. 2C). On the 14th day post-SCI, a time point at which NSCs have fully differentiated, we observed a higher density of neurons in the injured area of UTX KO mice (Fig. 2C, D).

#### In vitro analysis of SCMECs and NSCs differentiation

For in vitro experiments, SCMECs were isolated from both UTX KO and NC mice and identified via CD31 immunofluorescence (Fig. S2A). NSCs were isolated from WT mice and confirmed through Nestin and SOX2 immunofluorescence and differentiation ability (Fig. 2E, Fig. S2B). We then exposed NSCs to neuron-differentiation supernatant or astrocyte-differentiation supernatant from UTX KO and NC SCMECs cultures to assess their impact on NSCs differentiation. On the 5th day of intervention, we found that the proportion of Tuj-1^+^ (neuron marker) cells was higher, while the proportion of GFAP^+^ (astrocyte marker) cells was lower in the UTX KO SCMECs supernatant group (Fig. 2F-H). This suggested that the supernatant from UTX KO SCMECs promoted neural differentiation of NSCs in vitro.

### Elevated expression of L1CAM in UTX KO SCMECs

**Fig. 3:**
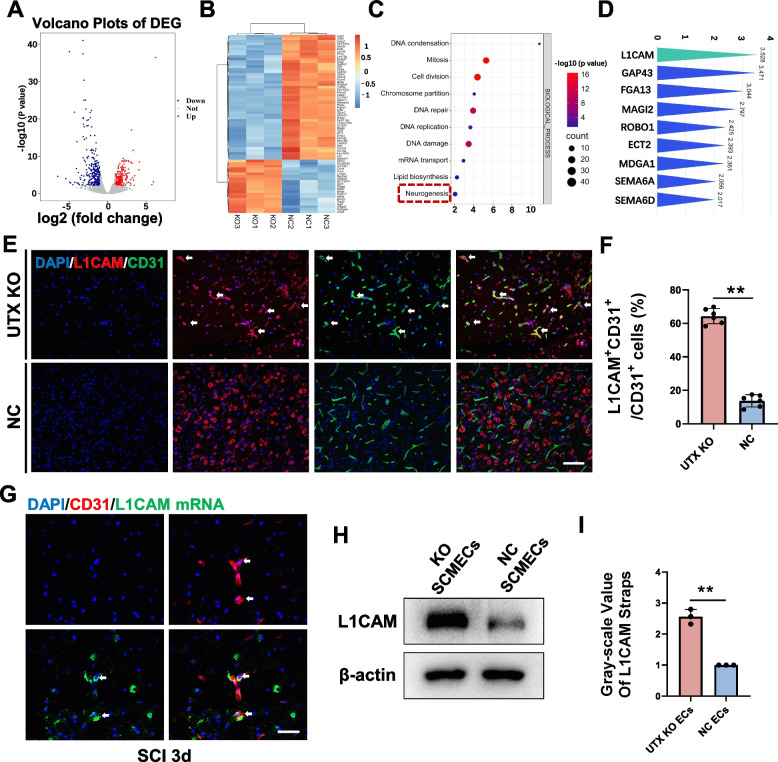
UTX KO SCMECs highly expressed L1CAM. **A** Volcano plot of differentially expressed mRNA in UTX KO SCMECs compared to NC SCMECs. **B** Hierarchical cluster heatmap of the differential mRNAs in UTX KO SCMECs compared to NC SCMECs. **C** Dot plot of GO enrichment analysis. **D** Fold change of neurogenesis related genes in SCMECs after UTX KO. **E** Immunofluorescent analysis of the expression of L1CAM (red) in UTX KO SCMECs (green) and NC SCMECs (green) on the 3rd day of SCI. Scale bar, 40 μm. **F** Statistical analysis of the ratio of L1CAM^+^CD31^+^ cells to CD31^+^ cells in figure E, *n* = 6 per group. **G** Fluorescence in situ hybridization analysis of the expression of L1CAM mRNA (green) in UTX KO SCMECs (red) on the 3rd day of SCI. Scale bar, 20 μm. **H** Western blotting analysis of the expression levels of the L1CAM proteins in UTX KO SCMECs and NC SCMECs. **I** Statistical analysis of L1CAM expression in each group in figure H, *n* = 3 per group. ^ns^*P* > 0.05, *P < 0.05, **P < 0.01, compared with corresponding control group

To elucidate the underlying mechanisms by which UTX KO in SCMECs influences NSCs differentiation, we conducted RNA sequencing on both UTX KO and NC SCMECs. Our analysis revealed a significant upregulation of the neurogenesis-related gene L1CAM in UTX KO SCMECs (Fig. 3A-D).

The L1CAM gene encodes an axonal glycoprotein that is a member of the immunoglobulin supergene family. L1CAM is critically involved in various aspects of nervous system development, including neural migration and differentiation. L1CAM is mainly expressed in neurons in CNS [43]. In the undamaged condition, L1CAM expression is low in endothelial cells. In cases of injury and inflammation, the expression of L1CAM increases in these cells [26]. We validated this through L1CAM immunofluorescence analysis. The expression of L1CAM is relatively low on SCMECS and NSCs in normal spinal cord of mice, and L1CAM is mainly expressed in neurons (Fig. S2C-H).

Because NSCs were in the process of differentiation on the 3rd day after SCI, we selected the 3rd day to observe the expression of L1CAM in SCMECs. Immunofluorescence and in situ hybridization assays corroborated the elevated expression of L1CAM in UTX KO SCMECs on the 3rd day post-SCI (Fig. 3E-G). Further in vitro validation using western blot analysis confirmed that L1CAM levels were significantly higher in UTX KO SCMECs compared to their NC counterparts (Fig. 3H, I).

### EVs secretion by SCMECs Post-SCI and its correlation with neural differentiation

#### SCMECs as secretory cells and EVs release post-SCI

**Fig. 4:**
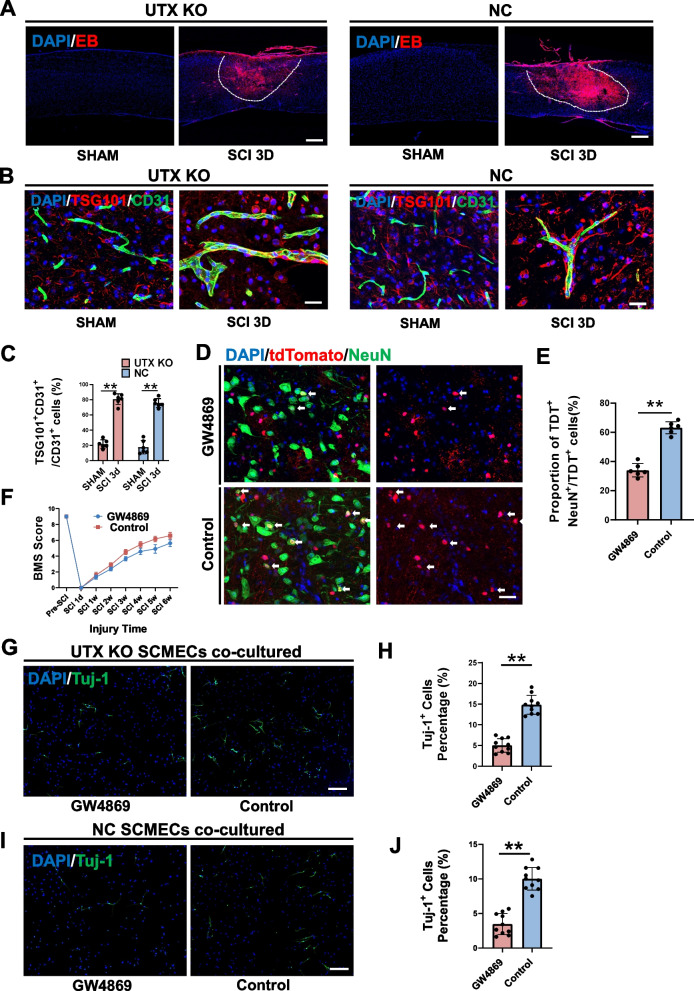
SCMECs were in the high EVs secretion state and Inhibition of EVs secretion hindered neural differentiation. **A** EB permeation assays of UTX KO mice and NC mice in the sham group and 3 days after SCI. Scale bar, 100 μm. **B** Immunofluorescent analysis of the expression of TSG101 (red) in UTX KO SCMECs (green) and NC SCMECs (green) in sham group and 3 days after SCI. Scale bar, 20 μm. **C** Statistical analysis of the ratio of TSG101^+^CD31^+^ cells to CD31^+^ cells in figure B, *n* = 6 per group. **D** The immunofluorescence analysis of the neural differentiation in NSCs lineage-traced mice 14 days post-SCI after GW4869 intervention. Scale bar, 20 μm. **E** Statistical analysis of the ratio of NeuN^+^tdtomato^+^ cells to tdTomato^+^ cells in figure D, *n* = 6 per group. **F** Distribution of the BMS scores over time post-SCI in GW4869 and Control groups. **G** Immunofluorescent analysis of the neural differentiation of NSCs co-cultured with UTX KO SCMECs intervened by GW4869 (GW4869 group) or PBS (Control group). Scale bar, 40 μm. **H** Statistical analysis of Tuj-1^+^ cells to all cells in each group in figure G, *n* = 10 per group. **I** Immunofluorescent analysis of the neural differentiation of NSCs co-cultured with NC SCMECs intervened by GW4869 or PBS (Control group). Scale bar, 40 μm. **J** Statistical analysis of Tuj-1^+^ cells to all cells in each group in figure I, *n* = 10 per group. ^ns^*P* > 0.05, *P < 0.05, **P < 0.01, compared with corresponding control group

To explore the communication mechanism between SCMECs and NSCs, we hypothesized that SCMECs may secrete specific substances to impact NSCs, given that vascular endothelial cells are inherently secretory [44]. SCI compromises the blood-spinal cord barrier, disrupting the plasma membrane integrity of SCMECs. This was confirmed through Evans blue (EB) permeation assays (Fig. 4A). Based on existing literature, cellular damage can stimulate EVs release as a membrane repair mechanism [27].

#### TSG101 as an indicator of EVs secretion

To assess whether damaged SCMECs are in a high EVs secretion state, we employed TSG101 as a reference marker, which is part of the ESCRT (endosomal sorting complex required for transport) complex and is positively correlated with EVs secretion [45–47]. Immunofluorescence revealed elevated TSG101 expression in UTX KO SCMECs and NC SCMECs on the 3rd day post-SCI compared to the respective sham group (Fig. 4B, C), suggesting increased EVs secretion after SCI.

#### Inhibition of EVs secretion impaired neural differentiation

To further investigate the role of EVs in NSCs differentiation, we administered GW4869 (2.5 μg/g, intraperitoneal injection) 1 h post-SCI to inhibit EVs secretion in NSCs lineage-traced mice. GW4869 is a specific inhibitor of membrane neutral sphingomyelinase (nSMase). It can inhibit ceramide mediated synthesis and release of EVs. The mechanism is that GW4869 prevents the hydrolysis of the membrane lipid sphingomyelin from producing bioactive lipid ceramide [48].The results showed reduced NeuN expression in tdTomato^+^ cells and lower Basso Mouse Scale (BMS) scores at various time points post-SCI in the GW4869 group (Fig. 4D-F), indicating that EVs secretion inhibition hampered neural differentiation and functional recovery.

#### Specific impact of SCMECs EVs on neural differentiation

To isolate the effects of EVs solely from SCMECs, we co-cultured UTX KO SCMECs or NC SCMECs with NSCs using a transwell chamber. EVs could traverse the membrane filter to reach the NSCs differentiation medium in the lower chamber. Inhibition of EVs secretion from UTX KO SCMECs using GW4869 led to a significant decrease in the proportion of Tuj-1^+^ NSCs compared to the control group (Fig. 4G, H). The use of GW4869 intervention in NC SCMEC also resulted in a significant decrease in the proportion of Tuj-1^+^ NSCs (Fig. 4I, J). These results confirmed that EVs from UTX KO SCMECs or NC SCMECs promoted neural differentiation.

### KO EVs facilitated neural differentiation post-SCI

#### L1CAM-loaded EVs as potential regulators of NSCs differentiation

As previously established, L1CAM, a gene implicated in neurogenesis, was significantly upregulated in UTX KO SCMECs. We hypothesized that the interaction between SCMECs and NSCs post-SCI might be mediated by the phagocytosis of SCMECs-derived EVs by NSCs, with L1CAM-loaded EVs modulating NSCs differentiation. This notion is supported by existing literature on the role of L1CAM-associated EVs in cellular communication [49].

#### Characterization of SCMECs-derived EVs

**Fig. 5:**
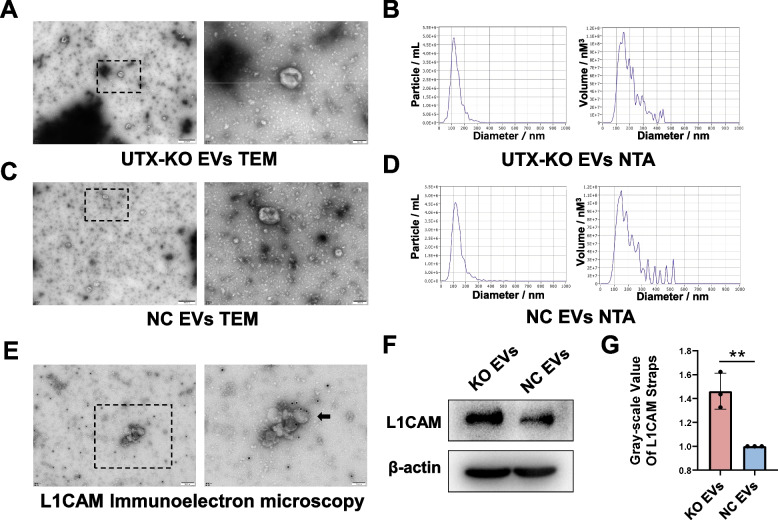
Characterization of SCMECs-Derived EVs and the Expression of L1CAM Protein in KO and NC EVs. **A** Scanning electron microscope view of UTX KO SCMECs-derived EVs (KO EVs). Scale bar, 500nn and 100nn. **B** Nanoparticle tracking analysis (NTA) to observe the size and distribution range of KO EVs. **C** Scanning electron microscope view of NC SCMECs-derived EVs (NC EVs). Scale bar, 500nn and 100nn. **D** Nanoparticle tracking analysis (NTA) to observe the size and distribution range of NC EVs. **E** Immunoelectron microscopy analysis of the distribution of L1CAM on KO EVs. Scale bar, 200nn and 100nn. **F** Western blotting analysis of the expression levels of the L1CAM proteins in KO EVs and NC EVs. **G** Statistical analysis of L1CAM expression in each group in figure F, *n* = 3 per group. ^ns^*P* > 0.05, *P < 0.05, **P < 0.01, compared with corresponding control group

We isolated EVs from both UTX KO and NC SCMECs and confirmed their identity through electron microscopy and nanoparticle tracking analysis (NTA) (Fig. 5A-D). Immunogold electron microscopy verified that these KO EVs expressed L1CAM, primarily localized to the membrane of EVs (Fig. 5E). Western blot analysis further revealed higher L1CAM expression in EVs from UTX KO SCMECs compared to NC SCMECs (Fig. 5F, G).

#### In vivo impact of EVs on NSCs differentiation

**Fig. 6:**
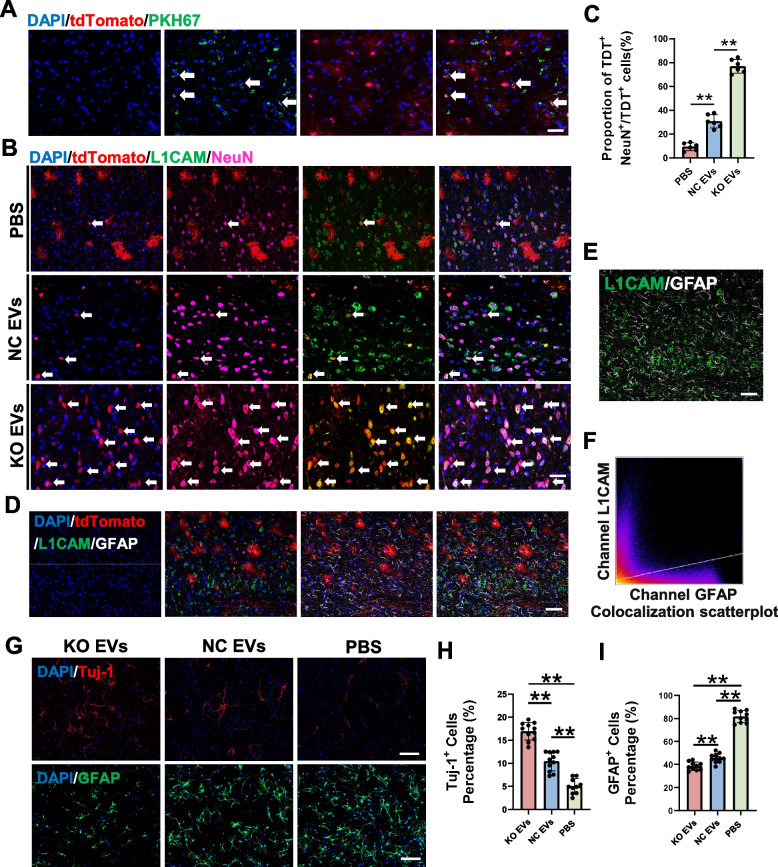
UTX KO SCMECs EVs promote neural differentiation. **A** Immunofluorescent identification of tdToamto^+^ NSCs (red) phagocytize KO EVs (green) labeld by PKH67. Scale bar, 40 μm. **B** Immunofluorescent analysis of the neural differentiation of tdToamto^+^ NSCs and expression of L1CAM in KO EVs group, NC EVs group and PBS group. Scale bar, 40 μm. **C** Statistical analysis of the ratio of NeuN^+^tdtomato^+^ cells to tdTomato^+^ cells in figure B, *n* = 6 per group. **D** Immunofluorescent analysis of the astrocytic differentiation of tdToamto^+^ NSCs and expression of L1CAM in PBS group. Scale bar, 40 μm. **E**, **F** colocalization analysis of GFAP channel and L1CAM channel in figure D. Scale bar, 40 μm. **G** Immunofluorescent analysis of the neural differentiation and astrocytic differentiation of NSCs in KO EVs group, NC EVs group and PBS group. Scale bar, 40 μm. **H**, **I** Statistical analysis of Tuj-1^+^ cells and GFAP^+^ cells to all cells in each group in figure G, *n* = 10 per group. ^ns^*P* > 0.05, *P < 0.05, **P < 0.01, compared with corresponding control group

We administered KO EVs and NC EVs to NSCs lineage-traced mice via tail vein injection at 1, 24, and 48 h post-SCI. EVs were labeled with the green fluorescent dye PHK67, confirming their uptake by tdTomato-labeled NSCs (Fig. 6A). To verify the safety of EVs in mice, we performed Hematoxylin & Eosin (HE) staining on various important organs of the mice on the 7th day post-SCI after EVs intervention according to the methods in the literature [50]. Compared with the control group, no significant abnormality was caused by the administration of KO EVs and NC EVs (Fig. S3A). Cells co-expressing tdTomato and L1CAM signals differentiated into NeuN-expressing neurons. The proportion of NSCs differentiating into neurons was significantly higher in the KO EVs group at 14 days post-SCI (Fig. 6B, C). Further analysis revealed that L1CAM-expressing NSCs did not differentiate into GFAP-expressing astrocytes (Fig. 6D-F).

#### In vitro validation of EVs-mediated neural differentiation

In vitro experiments employed KO EVs, NC EVs, and an equal volume of PBS to modulate NSCs differentiation. EVs uptake by NSCs was confirmed through PHK67 labeling (Fig. S3B). The proportion of Tuj-1^+^ cells was highest in the KO EVs group, followed by the NC EVs group, and lowest in the PBS group. Conversely, the proportion of GFAP^+^ cells was highest in the PBS group (Fig. 6G-I). These findings suggested that UTX KO SCMECs-derived EVs promoted NSCs differentiation into neurons, potentially due to their elevated L1CAM expression.

### KO EVs drived neural differentiation via the Akt/mTOR signaling pathway

#### Akt/mTOR signaling as a potential mechanism

To elucidate the mechanism by which KO EVs loaded with L1CAM regulate NSCs differentiation, we turned to existing literature. Previous studies have identified L1CAM as an activator of the Akt signaling pathway [51, 52], which is implicated in various biological processes including neural differentiation, axon specification, and synaptic plasticity [53]. The mammalian target of rapamycin (mTOR) is a key downstream effector of Akt in the CNS [54] and is part of a multifunctional protein complex involved in numerous physiological functions across various tissues [55].

#### Akt/mTOR activation and NSCs differentiation

**Fig. 7:**
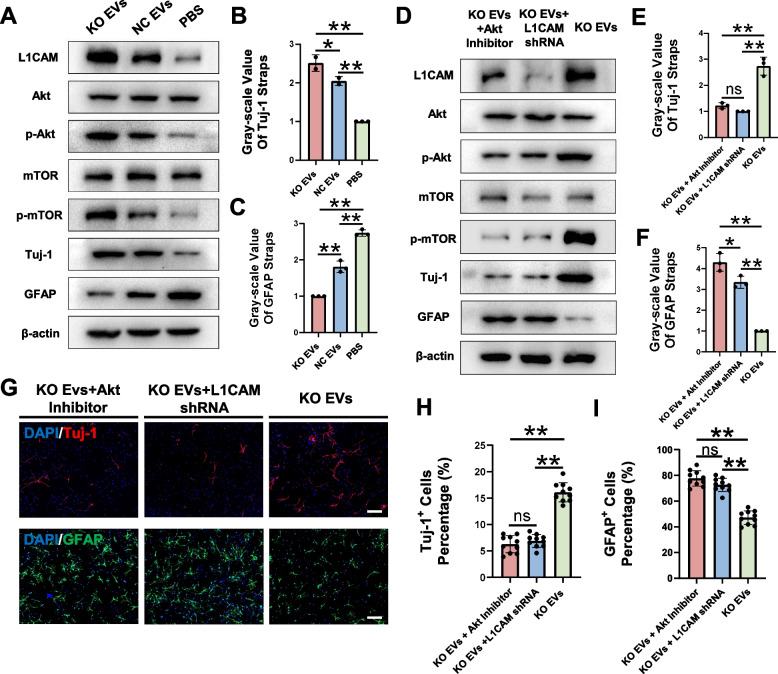
UTX KO SCMECs-derived EVs promotes neural differentiation through L1CAM-Akt/mTOR pathway. **A** Western blotting analysis of the expression levels of the L1CAM proteins, Akt/mTOR signaling pathway-related proteins, neuron marker protein Tuj-1 and astrocyte marker protein GFAP in KO EVs group, NC EVs group and PBS group. **B**, **C** Statistical analysis of Tuj-1 and GFAP expression in each group in figure A, *n* = 3 per group. **D** Western blotting analysis of the expression levels of the L1CAM proteins, Akt/mTOR signaling pathway-related proteins, Tuj-1 proteins and GFAP proteins in KO EVs plus Akt inhibitor intervention group NSCs, KO EVs plus L1CAM shRNA intervention group NSCs and KO EVs without other intervention group NSCs. **E**, **F** Statistical analysis of Tuj-1 and GFAP expression in each group in figure D, *n* = 3 per group. **G** Immunofluorescent analysis of the neural differentiation and astrocytic differentiation of NSCs in vitro in KO EVs plus Akt inhibitor intervention group NSCs, KO EVs plus L1CAM shRNA intervention group NSCs and KO EVs without other intervention group NSCs. Scale bar, 40 μm. **H**, **I** Statistical analysis of Tuj-1^+^ cells and GFAP^+^ cells to all cells in figure G, *n* = 10 per group. ^ns^*P* > 0.05, *P < 0.05, **P < 0.01, compared with corresponding control group

To investigate whether L1CAM-loaded EVs modulate NSCs differentiation via the Akt/mTOR pathway, we performed Western blot analysis on NSCs treated with KO EVs, NC EVs, and PBS (Fig. 7A). Our results revealed that KO EVs significantly elevated the levels of phosphorylated Akt and mTOR in NSCs. Moreover, the results of Western blot also indicate that KO EVs effectively promoted neurogenesis and suppressed astrogenesis in NSCs (Fig. 7B, C). These findings suggested that KO EVs, through their high L1CAM content, promoted NSCs differentiation into neurons by activating the Akt/mTOR signaling pathway.

#### Inhibition studies on Akt and L1CAM

To further clarify the role of Akt and mTOR phosphorylation in UTX KO EVs-L1CAM-mediated NSCs differentiation, we examined whether inhibiting the Akt pathway or knocking down L1CAM could reverse the neurogenesis induced by KO EVs. We employed the selective Akt inhibitor GSK690693 (MCE, HY-10249) and the recombinant adenovirus expressing L1CAM shRNA into our experimental setup. After allowing single cells digested from neurospheres to adhere, we added GSK690693, L1CAM shRNA, or PBS to the respective culture media. Western blot analysis showed that both Akt inhibition and L1CAM knockdown significantly reduced the levels of phosphorylated Akt and mTOR in NSCs (Fig. 7D). Additional western blot results (Fig. 7D-F) and immunofluorescence assays (Fig. 7G-I) indicated that inhibiting Akt or knocking down L1CAM effectively suppressed neurogenesis and activated astrogenesis in NSCs.

## Discussion

Our study observed notably low levels of neural differentiation post-SCI, underscoring a major challenge in recovery and treatment. Neural differentiation is a crucial process, significantly influencing SCI treatment outcomes. Our research highlights epigenetic regulation’s key role in SCMECs and NSCs communication. Specifically, SCMECs secrete EVs capable of regulating endogenous NSCs differentiation paths. This interaction is crucial, as it directly impacts nerve regeneration, vital for post-SCI recovery.

Consistent with previous research, our study reaffirms the dominance of astrocytic differentiation of endogenous NSCs post-SCI [56, 57]. This peaks around 2 weeks post-injury, coinciding with astrocytic glial scar formation, with NSCs contributing to about 30% of new astrocytes [58]. We also noted that by day 7 post-SCI, NSCs start differentiating in the injured area, comprising roughly 30% of total astrocytes.

One concerning observation is the near-complete depletion of endogenous NSCs as they differentiate into astrocytes [59]. This differentiation seems driven by an inhibitory glial environment, resulting from pathological events like cell death, ischemia, excitotoxicity, oedema, and immune responses at the injury site [5, 60]. Further complicating this are myelin-associated inhibitors (MAIs), which predominantly steer NSCs towards glial, not neuronal, differentiation [61, 62]. Glial scars, mainly formed by astrocytes, exacerbate this issue. They release CSPGs and myelin inhibitors, hindering neurogenesis via pathways like RhoA/ROCK and increased intracellular calcium [63–65]. This forms a negative feedback loop where NSCs’ glial differentiation contributes to scar formation, subsequently inhibiting their neural differentiation. Recent advancements suggest regulating endogenous NSCs to promote SCI repair [66]. Supporting this, Yang et al. showed that small molecule therapy can induce neurogenesis and inhibit astrogenesis in endogenous NSCs at injury sites, restoring neural function [67]. Deeper insights into the molecular mechanisms dictating NSCs differentiation can pave the way for improved regenerative therapies post-SCI.

The critical role of the Stem Cell Niche in cell activation, proliferation, and fate determination is undeniable [10, 68–70]. The distinct blood flow characteristics in these niches imply potential influences from blood-derived signals on NSCs [71, 72]. Factors secreted by endothelial cells, such as soluble amyloid precursor protein, show promise in regulating NSCs’ behavior [73, 74]. Post-CNS injury, there is a strong link between angiogenesis and neurogenesis, both aiding in functional recovery [9].

A major challenge in treating neurodegenerative diseases and CNS injuries is ineffective drug delivery, as most therapeutics cannot cross the blood–brain barrier effectively. EVs known to cross the blood–brain barrier [75], serve as crucial mediums for intercellular communication. Our research explored EVs’ potential as mediators of intercellular communication and as carriers for CNS injury treatments [76], supported by studies highlighting their role in tissue repair and regeneration [16, 77]. EVs from endothelial cells, particularly under stressful conditions like hypoxia, are intriguing research subjects, as hypoxia stimulates EV release [78, 79]. After SCI, the ischemic and hypoxic microenvironment, along with SCMEC membrane repair mechanisms, triggers an increase in EVs secretion by SCMECs. We suggest that these L1CAM-enriched EVs can boost NSCs’ neuronal differentiation, making them promising for SCI treatment [17, 80, 81]. L1CAM, overexpressed in gastric cancer and known to activate the Akt pathway, is significant in gastric cancer progression and metastasis [51]. Akt signalling pathway regulates diverse cellular activities, including proliferation, differentiation, and survival [53, 82, 83]. The ability of L1CAM to activate Akt and promote NSCs’ neural differentiation remains unclear. Our study found that knockdown of L1CAM or Akt pathway inhibition significantly reduces neural differentiation. These results imply that the Akt pathway is involved in L1CAM-promoted neural differentiation.

Additionally, EVs as non-cell therapeutics offer low immunogenicity, low tumorigenicity, and excellent biocompatibility [84, 85]. Our research also demonstrates that EVs can cross the blood-spinal cord barrier and reach the injury site safely and effectively, without significant organ damage. The therapeutic potential of EVs is supported by phase I clinical trials. In one trial, 24 volunteers showed good tolerance to nebulized mesenchymal stromal cells-derived EVs, with no serious adverse events reported up to 7 days post-nebulization [86]. Other studies confirm EVs’ role in enhancing neural differentiation [87, 88], and the regulation of endogenous NSCs for neurogenesis post-SCI is also validated [89]. Further research is required to translate these findings into practical CNS injury treatment methods.

While our focus was on NSCs differentiating into neurons and astrocytes, it’s crucial to recognize their potential to become oligodendrocytes. These cells are key in axon remyelination post-SCI [90], and their differentiation mechanisms warrant future investigation. Additionally, endothelial cells’ interactions with other cell types hint at more complex mechanisms, meriting further exploration for a deeper understanding.

## Conclusions

In conclusion, this study demonstrates that UTX deletion in SCMECs epigenetically regulates the neurogenesis of endogenous NSCs in the spinal cord through L1CAM. EVs mediate the communication between SCMECs and NSCs. Additionally, our findings indicate the role for the Akt/mTOR signalling pathways n UTX-KO- L1CAM-dependent neural differentiation.

### Supplementary Information

**Additional file 1: Figure S1.** Morphological changes of NSC in the central canal before and after SCI, spatial relationship between NSC and SCMECs, and differentiation of early stages of injury. **Figure S2.** Identification of SCMECs, the differentiation function of NSCs and the expression of L1CAM in normal spinal cord tissue. **Figure S3.** Hematoxylin & Eosin (HE) staining of various organs in mice and the uptake of EVs by NSCs in vitro.

**Additional file 2.**

## Data Availability

The datasets used and/or analysed during the current study are available from the corresponding author on reasonable request.

## Abbreviations

SCI: Spinal cord injury
UTX: Ubiquitously Transcribed tetratripeptide repeat on chromosome X
NSCs: Neural stem cells
UTX KO: UTX gene knockout
EVs: Extracellular vesicles
SVZ: Subventricular zone
SCMECs: Spinal cord microvascular endothelial cells
L1CAM: L1 cell adhesion molecule
NC: Negative control
mTOR: Mammalian target of rapamycin
CNS: Central nervous system

## References

[1] National Spinal Cord Injury Statistical Center (Birmingham, AL) (2016). Spinal Cord Injury (SCI) 2016 facts and figures at a glance. J Spinal Cord Med.

[2] Wu F, Liu L, Zhou H (2017). Endothelial cell activation in central nervous system inflammation. J Leukoc Biol.

[3] Fan B, Wei Z, Yao X, Shi G, Cheng X, Zhou X (2018). Microenvironment imbalance of spinal cord injury. Cell Transplant.

[4] Sabelstrom H, Stenudd M, Frisen J (2014). Neural stem cells in the adult spinal cord. Exp Neurol.

[5] Gregoire CA, Goldenstein BL, Floriddia EM, Barnabe-Heider F, Fernandes KJ (2015). Endogenous neural stem cell responses to stroke and spinal cord injury. Glia.

[6] Yang H, Lu P, McKay HM, Bernot T, Keirstead H, Steward O (2006). Endogenous neurogenesis replaces oligodendrocytes and astrocytes after primate spinal cord injury. J Neurosci.

[7] Yiu G, He Z (2006). Glial inhibition of CNS axon regeneration. Nat Rev Neurosci.

[8] Wurmser AE, Palmer TD, Gage FH (2004). Neuroscience. Cellular interactions in the stem cell niche. Science.

[9] Yang X-T, Bi Y-Y, Feng D-F (2011). From the vascular microenvironment to neurogenesis. Brain Res Bull.

[10] Tavazoie M, Van der Veken L, Silva-Vargas V, Louissaint M, Colonna L, Zaidi B (2008). A specialized vascular niche for adult neural stem cells. Cell Stem Cell.

[11] Shen Q, Goderie SK, Jin L, Karanth N, Sun Y, Abramova N (2004). Endothelial cells stimulate self-renewal and expand neurogenesis of neural stem cells. Science (New York, NY).

[12] Teng H, Zhang ZG, Wang L, Zhang RL, Zhang L, Morris D (2008). Coupling of angiogenesis and neurogenesis in cultured endothelial cells and neural progenitor cells after stroke. J Cereb Blood Flow Metab.

[13] Nakagomi N, Nakagomi T, Kubo S, Nakano-Doi A, Saino O, Takata M (2009). Endothelial cells support survival, proliferation, and neuronal differentiation of transplanted adult ischemia-induced neural stem/progenitor cells after cerebral infarction. Stem Cells (Dayton, Ohio).

[14] Jhas S, Ciura S, Belanger-Jasmin S, Dong Z, Llamosas E, Theriault FM (2006). Hes6 inhibits astrocyte differentiation and promotes neurogenesis through different mechanisms. J Neurosci.

[15] Ni S, Luo Z, Jiang L, Guo Z, Li P, Xu X (2019). UTX/KDM6A deletion promotes recovery of spinal cord injury by epigenetically regulating vascular regeneration. Mol Ther.

[16] Peng W, Xie Y, Luo Z, Liu Y, Xu J, Li C (2023). UTX deletion promotes M2 macrophage polarization by epigenetically regulating endothelial cell-macrophage crosstalk after spinal cord injury. J Nanobiotechnology.

[17] Nagaraj K, Hortsch M (2006). Phosphorylation of L1-type cell-adhesion molecules–ankyrins away!. Trends Biochem Sci.

[18] Li Y, Huang X, An Y, Ren F, Yang ZZ, Zhu H (2013). Cell recognition molecule L1 promotes embryonic stem cell differentiation through the regulation of cell surface glycosylation. Biochem Biophys Res Commun.

[19] Tsuru A, Mizuguchi M, Uyemura K, Takashima S (1996). Immunohistochemical expression of cell adhesion molecule L1 during development of the human brain. Early Hum Dev.

[20] Kenwrick S, Watkins A, De Angelis E (2000). Neural cell recognition molecule L1: relating biological complexity to human disease mutations. Hum Mol Genet.

[21] Dihné M, Bernreuther C, Sibbe M, Paulus W, Schachner M (2003). A new role for the cell adhesion molecule L1 in neural precursor cell proliferation, differentiation, and transmitter-specific subtype generation. J Neurosci.

[22] Cui YF, Hargus G, Xu JC, Schmid JS, Shen YQ, Glatzel M (2010). Embryonic stem cell-derived L1 overexpressing neural aggregates enhance recovery in Parkinsonian mice. Brain.

[23] Turner KN, Schachner M, Anderson RB (2009). Cell adhesion molecule L1 affects the rate of differentiation of enteric neurons in the developing gut. Dev Dyn.

[24] Jakovcevski I, Djogo N, Holters LS, Szpotowicz E, Schachner M (2013). Transgenic overexpression of the cell adhesion molecule L1 in neurons facilitates recovery after mouse spinal cord injury. Neuroscience.

[25] He X, Knepper M, Ding C, Li J, Castro S, Siddiqui M (2012). Promotion of spinal cord regeneration by neural stem cell-secreted trimerized cell adhesion molecule L1. PLoS One.

[26] Magrini E, Villa A, Angiolini F, Doni A, Mazzarol G, Rudini N (2014). Endothelial deficiency of L1 reduces tumor angiogenesis and promotes vessel normalization. J Clin Invest.

[27] Alves S, Pereira JM, Mayer RL, Goncalves ADA, Impens F, Cabanes D (2022). Cells responding to closely related cholesterol-dependent cytolysins release extracellular vesicles with a common proteomic content including membrane repair proteins. Toxins (Basel).

[28] Zhang YZ, Liu F, Song CG, Cao XL, Zhang YF, Wu HN (2018). Exosomes derived from human umbilical vein endothelial cells promote neural stem cell expansion while maintain their stemness in culture. Biochem Biophys Res Commun.

[29] Zhou S, Gao B, Sun C, Bai Y, Cheng D, Zhang Y (2020). Vascular endothelial cell-derived exosomes protect neural stem cells against ischemia/reperfusion injury. Neuroscience.

[30] Luga V, Zhang L, Viloria-Petit AM, Ogunjimi AA, Inanlou MR, Chiu E (2012). Exosomes mediate stromal mobilization of autocrine Wnt-PCP signaling in breast cancer cell migration. Cell.

[31] Valadi H, Ekstrom K, Bossios A, Sjostrand M, Lee JJ, Lotvall JO (2007). Exosome-mediated transfer of mRNAs and microRNAs is a novel mechanism of genetic exchange between cells. Nat Cell Biol.

[32] Liang B, Peng P, Chen S, Li L, Zhang M, Cao D (2013). Characterization and proteomic analysis of ovarian cancer-derived exosomes. J Proteomics.

[33] Lazar I, Clement E, Ducoux-Petit M, Denat L, Soldan V, Dauvillier S (2015). Proteome characterization of melanoma exosomes reveals a specific signature for metastatic cell lines. Pigment Cell Melanoma Res.

[34] Cau F, Fanni D, Manchia M, Gerosa C, Piras M, Murru R (2022). Expression of L1 Cell Adhesion Molecule (L1CAM) in extracellular vesicles in the human spinal cord during development. Eur Rev Med Pharmacol Sci.

[35] Ge X, Tang P, Rong Y, Jiang D, Lu X, Ji C (2021). Exosomal miR-155 from M1-polarized macrophages promotes EndoMT and impairs mitochondrial function via activating NF-κB signaling pathway in vascular endothelial cells after traumatic spinal cord injury. Redox Biol.

[36] He Z, Du J, Zhang Y, Xu Y, Huang Q, Zhou Q (2023). Kruppel-like factor 2 contributes to blood-spinal cord barrier integrity and functional recovery from spinal cord injury by augmenting autophagic flux. Theranostics.

[37] Dang G, Li T, Yang D, Yang G, Du X, Yang J (2022). T lymphocyte-derived extracellular vesicles aggravate abdominal aortic aneurysm by promoting macrophage lipid peroxidation and migration via pyruvate kinase muscle isozyme 2. Redox Biol.

[38] Njock M-S, O’Grady T, Nivelles O, Lion M, Jacques S, Cambier M (2022). Endothelial extracellular vesicles promote tumour growth by tumour-associated macrophage reprogramming. J Extracell Vesicles.

[39] Basso DM, Fisher LC, Anderson AJ, Jakeman LB, McTigue DM, Popovich PG (2006). Basso mouse scale for locomotion detects differences in recovery after spinal cord injury in five common mouse strains. J Neurotrauma.

[40] Shimada IS, Acar M, Burgess RJ, Zhao Z, Morrison SJ (2017). Prdm16 is required for the maintenance of neural stem cells in the postnatal forebrain and their differentiation into ependymal cells. Genes Dev.

[41] Xue X, Shu M, Xiao Z, Zhao Y, Li X, Zhang H (2022). Lineage tracing reveals the origin of Nestin-positive cells are heterogeneous and rarely from ependymal cells after spinal cord injury. Sci China Life Sci.

[42] Ohab JJ, Fleming S, Blesch A, Carmichael ST (2006). A neurovascular niche for neurogenesis after stroke. J Neuroscience.

[43] Burden-Gulley SM, Pendergast M, Lemmon V (1997). The role of cell adhesion molecule L1 in axonal extension, growth cone motility, and signal transduction. Cell Tissue Res.

[44] Freyer D, Manz R, Ziegenhorn A, Weih M, Angstwurm K, Docke WD (1999). Cerebral endothelial cells release TNF-alpha after stimulation with cell walls of Streptococcus pneumoniae and regulate inducible nitric oxide synthase and ICAM-1 expression via autocrine loops. J Immunol.

[45] Hessvik NP, Llorente A (2018). Current knowledge on exosome biogenesis and release. Cell Mol Life Sci.

[46] Colombo M, Moita C, van Niel G, Kowal J, Vigneron J, Benaroch P (2013). Analysis of ESCRT functions in exosome biogenesis, composition and secretion highlights the heterogeneity of extracellular vesicles. J Cell Sci.

[47] Yan C, Tian X, Li J, Liu D, Ye D, Xie Z (2021). A high-fat diet attenuates AMPK alpha1 in adipocytes to induce exosome shedding and nonalcoholic fatty liver development in vivo. Diabetes.

[48] Catalano M, O’Driscoll L (2020). Inhibiting extracellular vesicles formation and release: a review of EV inhibitors. J Extracell Vesicles.

[49] Gomes DE, Witwer KW (2022). L1CAM-associated extracellular vesicles: a systematic review of nomenclature, sources, separation, and characterization. J Extracell Biol.

[50] Xu J, Shi C, Yuan F, Ding Y, Xie Y, Liu Y (2024). Targeted transplantation of engineered mitochondrial compound promotes functional recovery after spinal cord injury by enhancing macrophage phagocytosis. Bioact Mater.

[51] Chen DL, Zeng ZL, Yang J, Ren C, Wang DS, Wu WJ (2013). L1cam promotes tumor progression and metastasis and is an independent unfavorable prognostic factor in gastric cancer. J Hematol Oncol.

[52] Zhang LY, Shen ZX, Guo L (2022). Inhibiting L1CAM reverses cisplatin resistance of triple negative breast cancer cells by blocking AKT signaling pathway. Cancer Invest.

[53] Manning BD, Cantley LC (2007). AKT/PKB signaling: navigating downstream. Cell.

[54] Manning BD, Toker A (2017). AKT/PKB signaling: navigating the network. Cell.

[55] Laplante M, Sabatini DM (2012). mTOR signaling in growth control and disease. Cell.

[56] Sellers DL, Maris DO, Horner PJ (2009). Postinjury niches induce temporal shifts in progenitor fates to direct lesion repair after spinal cord injury. J Neurosci.

[57] Horky LL, Galimi F, Gage FH, Horner PJ (2006). Fate of endogenous stem/progenitor cells following spinal cord injury. J Comp Neurol.

[58] McTigue DM, Sahinkaya FR (2011). The fate of proliferating cells in the injured adult spinal cord. Stem Cell Res Ther.

[59] Li X, Floriddia EM, Toskas K, Fernandes KJL, Guérout N, Barnabé-Heider F (2016). Regenerative potential of ependymal cells for spinal cord injuries over time. EBioMedicine.

[60] Barnabé-Heider F, Frisén J (2008). Stem cells for spinal cord repair. Cell Stem Cell.

[61] Wang B, Xiao Z, Chen B, Han J, Gao Y, Zhang J (2008). Nogo-66 promotes the differentiation of neural progenitors into astroglial lineage cells through mTOR-STAT3 pathway. PLoS One.

[62] Hofstetter CP, Holmström NAV, Lilja JA, Schweinhardt P, Hao J, Spenger C (2005). Allodynia limits the usefulness of intraspinal neural stem cell grafts; directed differentiation improves outcome. Nat Neurosci.

[63] Winton MJ, Dubreuil CI, Lasko D, Leclerc N, McKerracher L (2002). Characterization of new cell permeable C3-like proteins that inactivate Rho and stimulate neurite outgrowth on inhibitory substrates. J Biol Chem.

[64] Hasegawa Y, Fujitani M, Hata K, Tohyama M, Yamagishi S, Yamashita T (2004). Promotion of axon regeneration by myelin-associated glycoprotein and Nogo through divergent signals downstream of Gi/G. J Neurosci.

[65] Rudge JS, Silver J (1990). Inhibition of neurite outgrowth on astroglial scars in vitro. J Neurosci.

[66] Gilbert EAB, Lakshman N, Lau KSK, Morshead CM (2022). Regulating endogenous neural stem cell activation to promote spinal cord injury repair. Cells.

[67] Yang Y, Fan Y, Zhang H, Zhang Q, Zhao Y, Xiao Z (2021). Small molecules combined with collagen hydrogel direct neurogenesis and migration of neural stem cells after spinal cord injury. Biomaterials.

[68] Mirzadeh Z, Merkle FT, Soriano-Navarro M, Garcia-Verdugo JM, Alvarez-Buylla A (2008). Neural stem cells confer unique pinwheel architecture to the ventricular surface in neurogenic regions of the adult brain. Cell Stem Cell.

[69] Shen Q, Wang Y, Kokovay E, Lin G, Chuang S-M, Goderie SK (2008). Adult SVZ stem cells lie in a vascular niche: a quantitative analysis of niche cell-cell interactions. Cell Stem Cell.

[70] Andreotti JP, Silva WN, Costa AC, Picoli CC, Bitencourt FCO, Coimbra-Campos LMC (2019). Neural stem cell niche heterogeneity. Semin Cell Dev Biol.

[71] Lacar B, Young SZ, Platel J-C, Bordey A (2011). Gap junction-mediated calcium waves define communication networks among murine postnatal neural progenitor cells. Eur J Neurosci.

[72] Culver JC, Vadakkan TJ, Dickinson ME (2013). A specialized microvascular domain in the mouse neural stem cell niche. PLoS One.

[73] Azevedo PO, Lousado L, Paiva AE, Andreotti JP, Santos GSP, Sena IFG (2017). Endothelial cells maintain neural stem cells quiescent in their niche. Neuroscience.

[74] Sato Y, Uchida Y, Hu J, Young-Pearse TL, Niikura T, Mukouyama Y-S (2017). Soluble APP functions as a vascular niche signal that controls adult neural stem cell number. Development.

[75] Yang HC, Ham YM, Kim JA, Rhee WJ (2021). Single-step equipment-free extracellular vesicle concentration using super absorbent polymer beads. J Extracell Vesicles.

[76] Rufino-Ramos D, Albuquerque PR, Carmona V, Perfeito R, Nobre RJ, Pereira de Almeida L (2017). Extracellular vesicles: novel promising delivery systems for therapy of brain diseases. J Control Release.

[77] Dutta D, Khan N, Wu J, Jay SM (2021). Extracellular vesicles as an emerging frontier in spinal cord injury pathobiology and therapy. Trends Neurosci.

[78] Ludwig N, Yerneni SS, Menshikova EV, Gillespie DG, Jackson EK, Whiteside TL (2020). Simultaneous inhibition of glycolysis and oxidative phosphorylation triggers a multi-fold increase in secretion of exosomes: possible role of 2'3'-cAMP. Sci Rep.

[79] Tse SW, Tan CF, Park JE, Gnanasekaran J, Gupta N, Low JK (2020). Microenvironmental hypoxia induces dynamic changes in lung cancer synthesis and secretion of extracellular vesicles. Cancers (Basel).

[80] Kiss JZ, Muller D (2001). Contribution of the neural cell adhesion molecule to neuronal and synaptic plasticity. Rev Neurosci.

[81] Tsuchimochi R, Yamagami K, Kubo N, Amimoto N, Raudzus F, Samata B (2023). Viral delivery of L1CAM promotes axonal extensions by embryonic cerebral grafts in mouse brain. Stem Cell Reports.

[82] Read DE, Gorman AM (2009). Involvement of Akt in neurite outgrowth. Cell Mol Life Sci.

[83] Kim TH, Sung SE, Cheal Yoo J, Park JY, Yi GS, Heo JY (2018). Copine1 regulates neural stem cell functions during brain development. Biochem Biophys Res Commun.

[84] Somiya M, Yoshioka Y, Ochiya T (2018). Biocompatibility of highly purified bovine milk-derived extracellular vesicles. J Extracell Vesicles.

[85] Jia Y, Yu L, Ma T, Xu W, Qian H, Sun Y (2022). Small extracellular vesicles isolation and separation: current techniques, pending questions and clinical applications. Theranostics.

[86] Shi M-M, Yang Q-Y, Monsel A, Yan J-Y, Dai C-X, Zhao J-Y (2021). Preclinical efficacy and clinical safety of clinical-grade nebulized allogenic adipose mesenchymal stromal cells-derived extracellular vesicles. J Extracell Vesicles.

[87] Ditte Z, Silbern I, Ditte P, Urlaub H, Eichele G (2022). Extracellular vesicles derived from the choroid plexus trigger the differentiation of neural stem cells. J Extracell Vesicles.

[88] Esteves M, Abreu R, Fernandes H, Serra-Almeida C, Martins PAT, Barão M (2022). MicroRNA-124-3p-enriched small extracellular vesicles as a therapeutic approach for Parkinson’s disease. Mol Ther.

[89] Li J, Luo W, Xiao C, Zhao J, Xiang C, Liu W (2023). Recent advances in endogenous neural stem/progenitor cell manipulation for spinal cord injury repair. Theranostics.

[90] Llorens-Bobadilla E, Chell JM, Le Merre P, Wu Y, Zamboni M, Bergenstrahle J (2020). A latent lineage potential in resident neural stem cells enables spinal cord repair. Science.

