# Supplementary figures and images for: Extracellular vesicles from UTX-knockout endothelial cells boost neural stem cell differentiation in spinal cord injury
Source: Cell Commun Signal. 2024 Feb 29;22:155. doi: 10.1186/s12964-023-01434-4 (PMC10903014; doi:10.1186/s12964-023-01434-4)

# A DAPI/**tdTomato**/Nestin

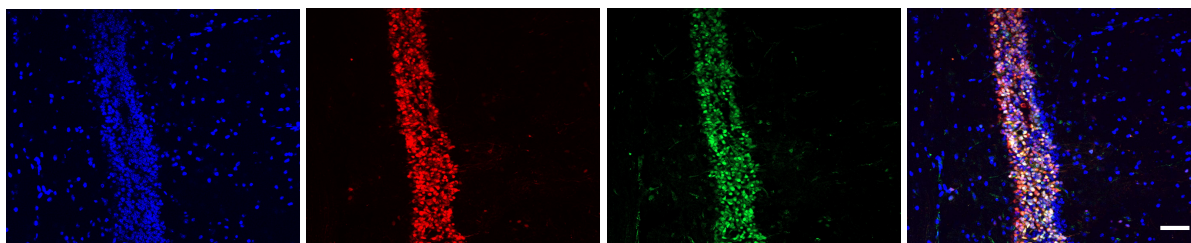

# B

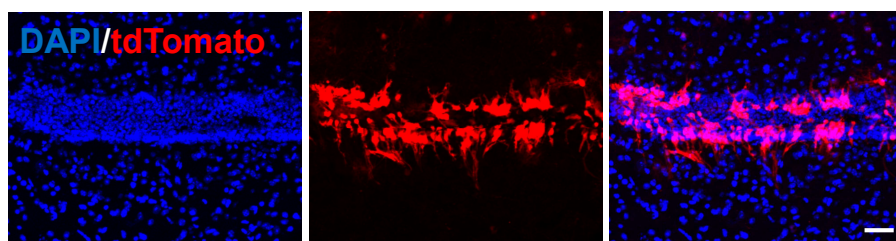

# C

## DAPI/**tdToamto**/CD31

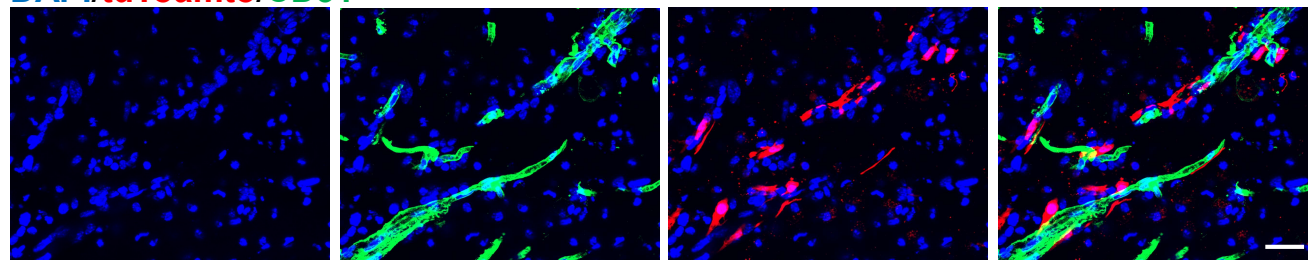

# D

SCI 3D

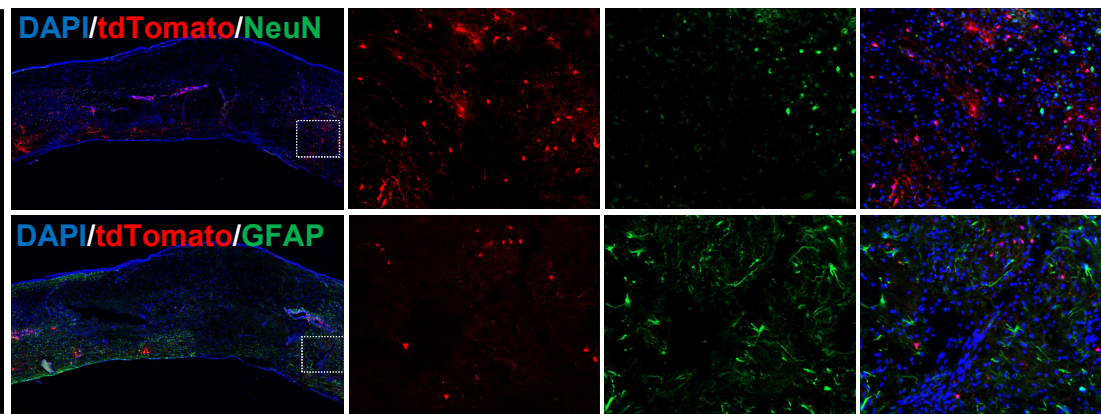

SCI 7D

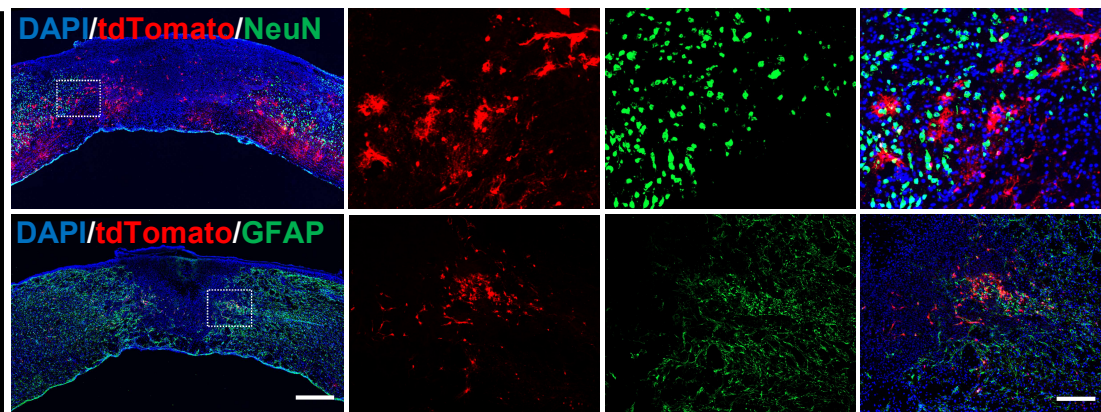

# E

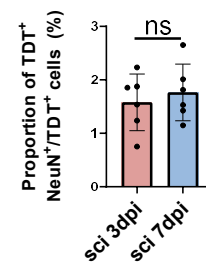

# F

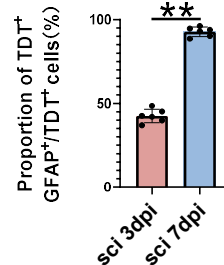

# G

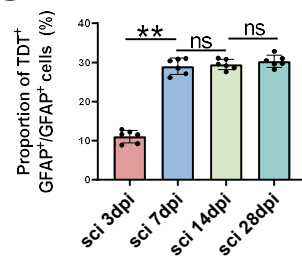

Supplement: Supplementary file 2 — Additional file 1: Figure S1. Morphological changes of NSC in the central canal before and after SCI, spatial relationship between NSC and SCMECs, and differentiation of early stages of injury. Figure S2. Identification of SCMECs, the differentiation function of NSCs and the expression of L1CAM in normal spinal cord tissue. Figure S3. Hematoxylin & Eosin (HE) staining of various organs in mice and the uptake of EVs by NSCs in vitro. [file 12964_2023_1434_MOESM1_ESM.zip › Figure S1_ESM.pdf]

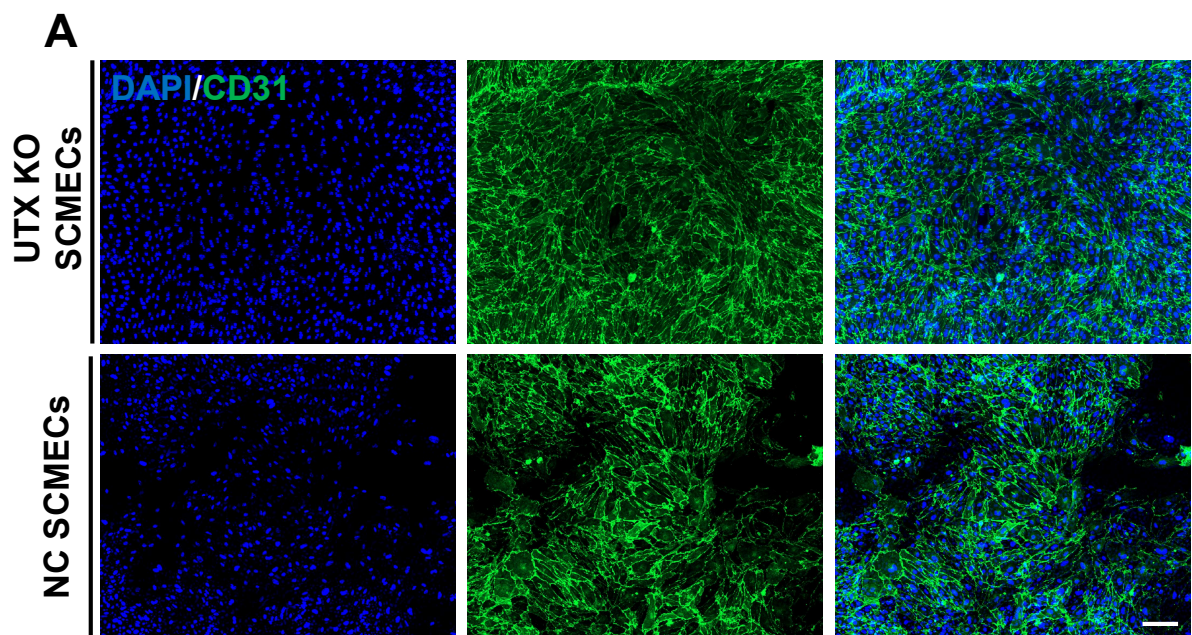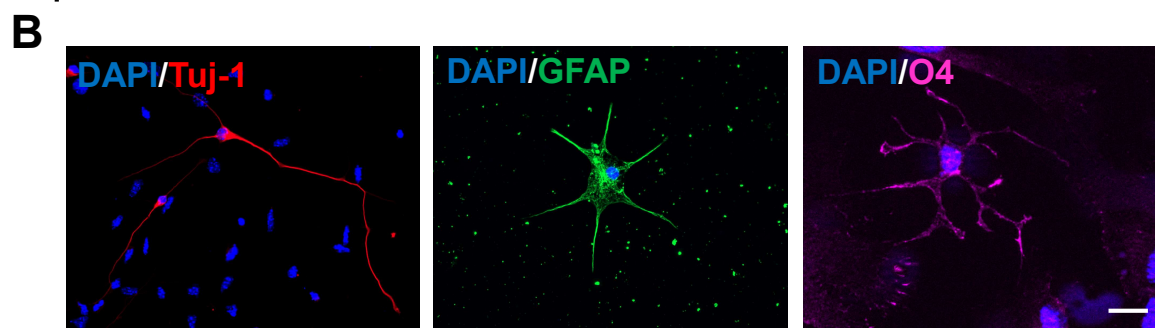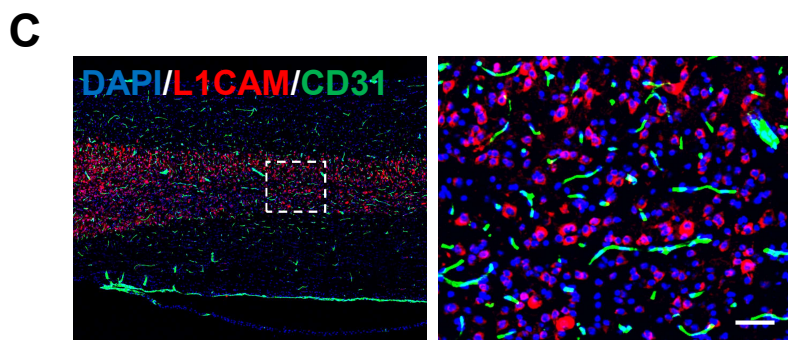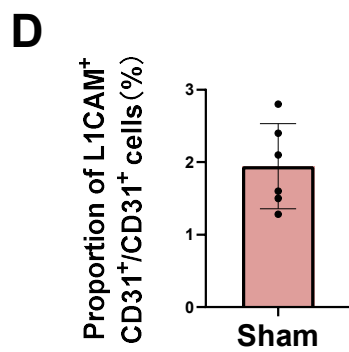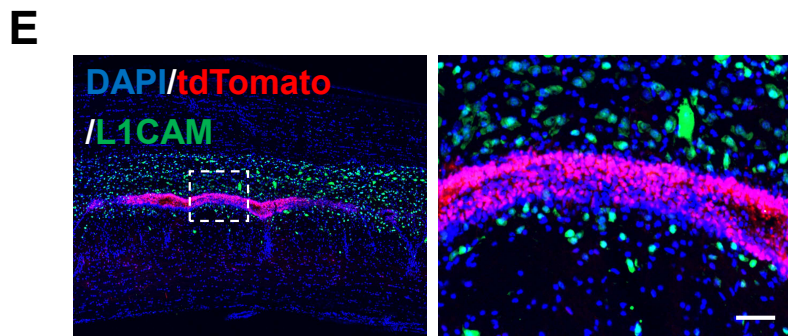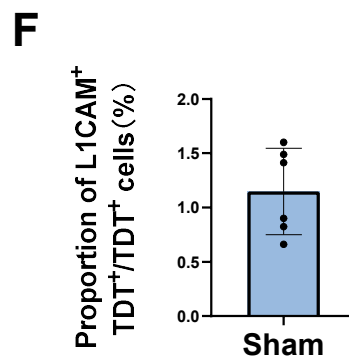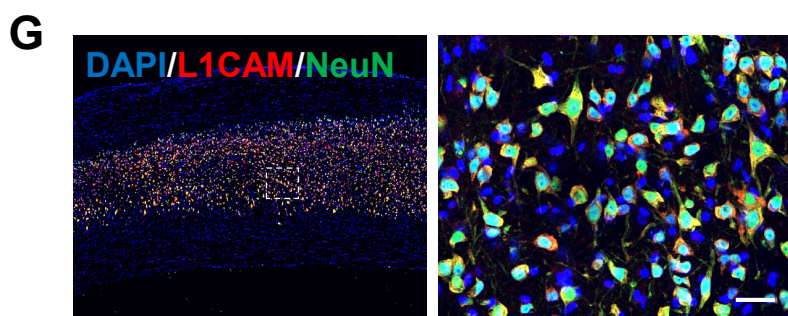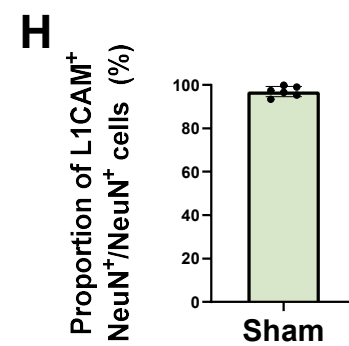

Supplement: Supplementary file 2 — Additional file 1: Figure S1. Morphological changes of NSC in the central canal before and after SCI, spatial relationship between NSC and SCMECs, and differentiation of early stages of injury. Figure S2. Identification of SCMECs, the differentiation function of NSCs and the expression of L1CAM in normal spinal cord tissue. Figure S3. Hematoxylin & Eosin (HE) staining of various organs in mice and the uptake of EVs by NSCs in vitro. [file 12964_2023_1434_MOESM1_ESM.zip › Figure S2_ESM.pdf]

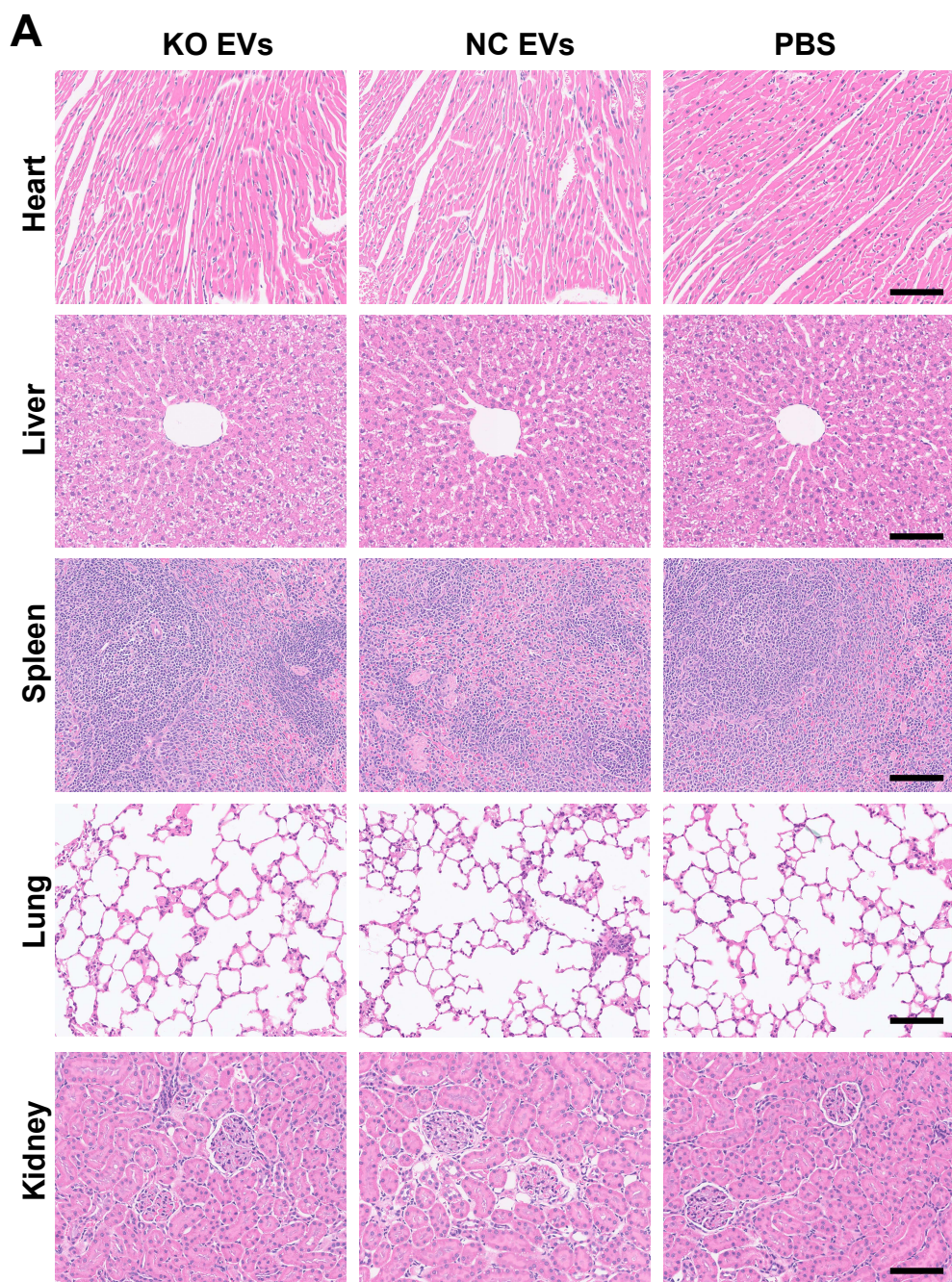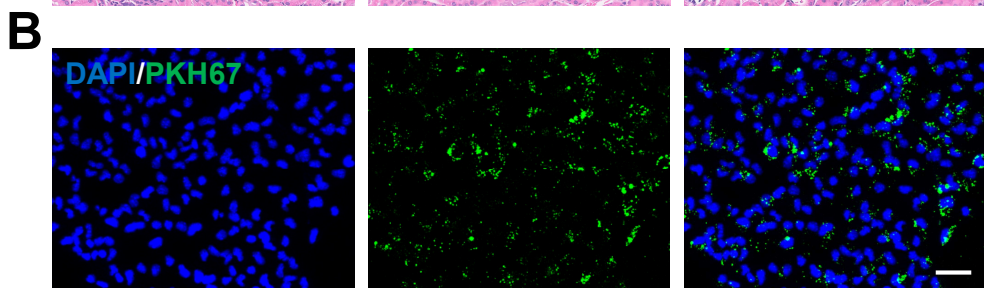

Supplement: Supplementary file 2 — Additional file 1: Figure S1. Morphological changes of NSC in the central canal before and after SCI, spatial relationship between NSC and SCMECs, and differentiation of early stages of injury. Figure S2. Identification of SCMECs, the differentiation function of NSCs and the expression of L1CAM in normal spinal cord tissue. Figure S3. Hematoxylin & Eosin (HE) staining of various organs in mice and the uptake of EVs by NSCs in vitro. [file 12964_2023_1434_MOESM1_ESM.zip › Figure S3_ESM.pdf]

**Fig 3H**

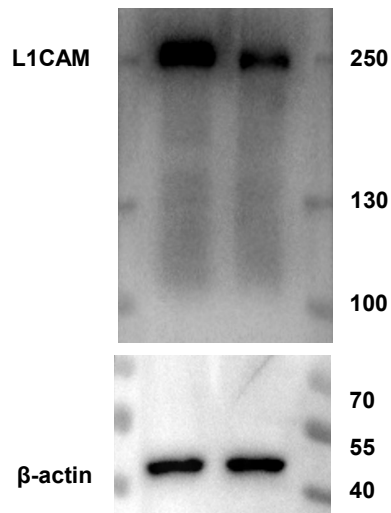

**Fig 5F**

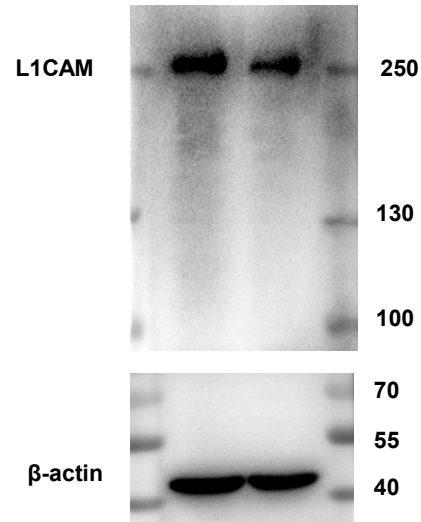

**Fig 7A**

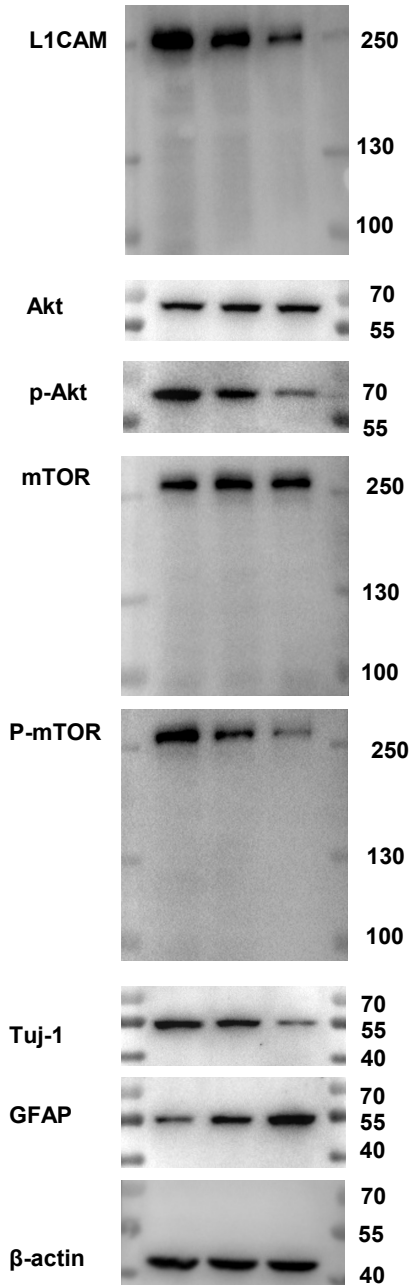

**Fig 7D**

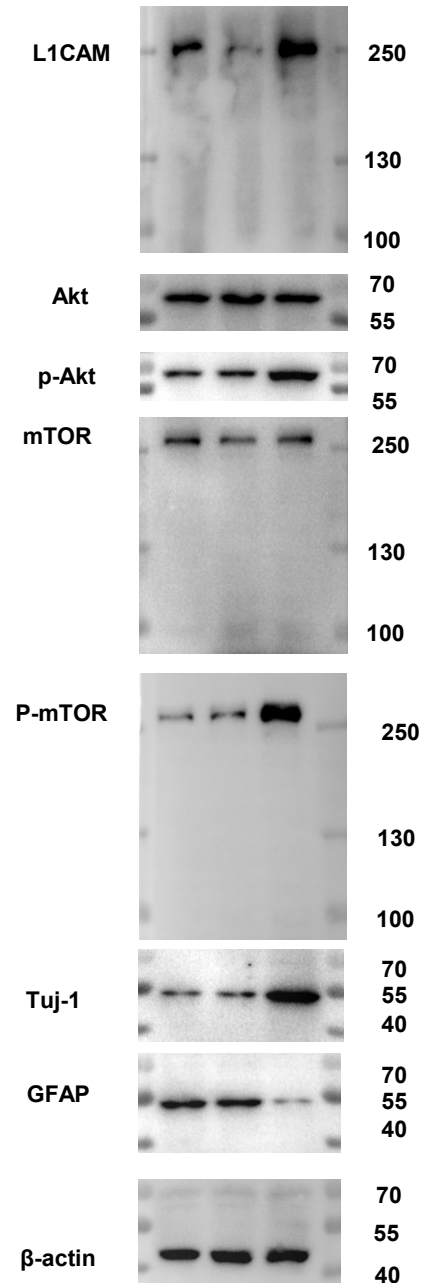

Supplement: Supplementary file 3 — Additional file 2. [file 12964_2023_1434_MOESM2_ESM.pdf]
